# Mitochondrial Function in Neurons and Glia in Health and Its Alteration in Parkinson's Disease: A Review

**DOI:** 10.2174/011570159X415585260303184042

**Published:** 2026-05-15

**Authors:** Monika Kaushik, Mrugendra Potdar, Prathap Madeswaraguptha, Murugesan Vanangamudi, Md Abubakar, Manoj Kumar Mishra, Sachchida Nand Rai

**Affiliations:** 1 Department of Pharmacology, Amity Institute of Pharmacy, Amity University, Gwalior, India; Current Affiliation: School of Medical & Allied Sciences, K R Mangalam University, Gurugram, Haryana, 122103, India;; 2 Department of Pharmaceutics, Shri Vile Parke Kelavani Mandal’s Institute of Pharmacy, Dhule, Maharashtra, India;; 3 Department of Pharmaceutical Sciences, School of Biotechnology and Pharmaceutical Sciences, Vignan’s Foundation for Science, Technology and Research (Deemed to be University), Guntur, India;; 4 Institute of Pharmaceutical Research, GLA University, Mathura, Uttar Pradesh, 281406, India;; 5 Department of Pharmacology and Toxicology, National Institution of Pharmaceutical Education and Research, Hajipur, Vaishali, Bihar, 844102, India;; 6 Department of Pharmaceutics, Amity Institute of Pharmacy, Amity University, Gwalior, Madhya Pradesh, India;; 7 Centre of Experimental Medicine & Surgery, Institute of Medical Sciences, Banaras Hindu University, Varanasi, India; Current Affiliation: University Centre for Research & Development (UCRD), Chandigarh University, Mohali, Punjab, 140413, India

**Keywords:** Astrocytes, calcium ions, free fatty acid oxidation, glycolysis, microglia, mitochondria, neuroinflammation, neurons, oligodendrocytes, oxidative stress, Parkinson’s disease

## Abstract

**Introduction:**

Mitochondria play an important role in maintaining redox balance, energy, calcium, and the viability of neurons. The mitochondrial dysfunction is one of the primary sources of glial activation and dopaminergic neuron loss in Parkinson's disease (PD). The key biochemical elements of the pathogenesis of PD include impaired oxidative phosphorylation, elevated generation of reactive oxygen species (ROS), and impaired mitophagy.

**Methods:**

This review is a synthesis and stringent evaluation of recent experimental, clinical and genetic studies relating mitochondrial dysfunction and Parkinson's disease (PD). We examined information on bioenergetics, mitochondrial dynamics, calcium homeostasis, and interactions between neurons and glia. The molecular and therapeutic importance of therapies, such as mitophagy modulators, bioenergetic enhancers, and mitochondrial antioxidants, was investigated.

**Results:**

The absence of Complex I, excess ROS, mitochondrial DNA damage, and nonfunctioning fusion-fission cycles leads to neurodegeneration. The glial metabolic abnormalities worsen the oxidative stress and neuroinflammation, weakening the support of the neurons. The effects of impaired mitophagy are the accumulation of dysfunctional mitochondria, and the effects of calcium overload disrupt energy metabolism. Neuroprotective effects of such substances as spermidine, urolithin A, resveratrol, α-lipoic acid, MitoQ, SkQ1, or CoQ10 have been shown using preclinical research. Sacrifices such as exercising and proper dieting enable the mitochondria to perform better and become stronger.

**Discussion:**

Mitochondrial dysfunction enhances the progression of PD through oxidative stress, bioenergetic breakdown, and inflammatory signalling. Attention to these related systems is an entire way to alter the direction of a disease.

**Conclusion:**

PD can be treated using an increase in mitochondrial quality control, redox regulation, and metabolic efficiency. Continued studies in the framework of precision medicine are required to validate the safety and effectiveness of mitochondrial-targeted medications.

## 1 INTRODUCTION

### 1.1 Role of Mitochondria in Cellular and Brain Function

 Mitochondria are bicellular organelles that contain two membranes, which play a critical role in energy production and calcium storage as well as regulating the reactive oxygen species (ROS) [1, 2]. Synaptic transmission, axonal transport, and plasticity in neurons would not be possible without mitochondrial activity, which has extremely high metabolic demands [3]. The brain is less than a quarter of the total mass of the body, but it consumes nearly a quarter of the glucose in the body. This demonstrates the fact that it is essential that the mitochondria should be well-functioning to produce ATP by oxidative phosphorylation [4]. Substrates are converted to ATP by the mitochondria, which uses the electron transport chain to transfer the electrons, and the proton gradient is formed across the inner mitochondrial membrane. It is a life process that must happen; however, it also generates reactive oxygen species (ROS), such as superoxide and hydrogen peroxide. In excess production, ROS may lead to oxidative stress, which is a potential cause of damage to the mitochondrial DNA, lipids, and proteins [7, 8]. This form of injury is particularly susceptible to neurons since they do not divide and require considerable amounts of mitochondrial energy [5]. The common features of aging are mitochondrial dysfunction and changes in energy metabolism, which are becoming part of the etiology of neurodegenerative diseases, such as Alzheimer's disease (AD), Parkinson's disease (PD), and amyotrophic lateral sclerosis (ALS). Impaired mitochondrial biosynthesis, impaired mitophagy, and changes in mitochondrial dynamics (fission and fusion) can lead to these dysfunctions. As a result, the energy deficiency, increased oxidative stress, and calcium imbalance can trigger the neuronal injury and progressive degeneration [6].

#### 1.1.1 Neurodegeneration, Metabolic Adaptation, and Hormesis

 Metabolic changes that disrupt neuronal homeostasis significantly influence neurodegenerative disorders. The concept of hormesis, *i.e*., small doses of stress (caloric restriction/physical exercise) that result in adaptive, protective mechanisms in the brain, has emerged as an important cornerstone in understanding how the brain adapts to stress [7]. Hormetic effects can include the stimulation of antioxidant mechanisms, mitochondrial efficiency, or protein homeostasis, all of which help delay neuronal aging and dementia. Mitochondrial dysfunctions are especially vulnerable to the cerebral cortex, whose functioning is highly dependent on the well-regulated production of energy. The damage of cortical networks may be caused by metabolic inflexibility and impaired adaptive mechanisms, which worsen cognitive impairments in neurodegenerative diseases [8].

#### 1.1.2 Mitochondrial Network Dynamics in Neurons

 Neurons require a well-structured and sensitive mitochondrial network to alter in shape and location depending on the amount of energy required [9]. Fission, fusion, biogenesis, and transport are processes that regulate mitochondrial dynamics, which ensure that energy is distributed evenly across axons and dendrites. The dynamics of these changes can alter the level of neuronal stimulation, axonal regeneration, and neurotransmission. Furthermore, the violations of ROS and calcium homeostasis may disrupt the network and cause increased fragmentation and malfunctioning of mitochondria. An important indicator of most neurodegenerative diseases is the loss of the ability to maintain mitochondrial networks in a dynamic balance. The mitochondrial structural distortions are often associated with a reduction in respiratory efficiency and impaired ATP production, which increases the neuronal stress and loss [10].

#### 1.1.3 Mitochondria, Oxidative Stress, and Neuroinflammation

Mitochondria not only generate energy; they are also signaling centers, particularly in immunological responses in the brain. High levels of mitochondrial reactive oxygen species (ROS) are capable of triggering the innate immune system, leading to neuroinflammatory cascades [11]. Particularly, this is significant in PD, where oxidative damage and mitochondrial dysfunction are exacerbated by the activation of microglia and the production of pro-inflammatory cytokines (such as TNF-α and IL-6). Recent studies support a two-way relationship between neuroinflammation and mitochondrial dysfunction. Proteins such as Parkin and PINK1 that participate in mitochondrial quality control not only regulate mitophagy but also alter damage-associated molecular patterns (DAMP) release, initiating inflammatory pathways [12]. Disruption of such mechanisms as observed in PD results in a vicious circle of inflammation and mitochondrial harm.

### 1.2 Role of Mitochondrial Calcium and Oxidative Stress in Neurodegenerative Disease Mechanisms

Mitochondria play a role in maintaining the calcium (Ca^2+^) levels within the cells. They achieve this through buffering temporary changes in the cytosolic Ca^2+^ and modulating the action of various enzymes of metabolism. In the usual conditions, Ca^2+^ is rapidly absorbed in the cytosol through mitochondria, particularly during neuronal activity, to regulate ATP production and maintain a steady energy level. But, perturbations of this highly tuned process may have undesirable outcomes, such as apoptosis and necrosis [13]. It is known that calcium overload in mitochondria is one of the stimulators of the opening of the mitochondrial permeability transition pore (mPTP), which is a vital step in apoptosis. Increased Ca^2+^ levels in mitochondria alter the membrane potential (DPsm), lower the effectiveness of oxidative phosphorylation, and promote the release of pro-apoptotic proteins like cytochrome c. In addition to this, elevated Ca^2+^ enhances the production of ROS through the perturbation of the electron transport chain and thus worsens oxidative stress and cell damage [14].

When the level of ROS exceeds the amount of antioxidants, this may lead to oxidative stress, which may cause damage to proteins, lipids, and DNA. The primary source of ROS and the primary location of its disposal are the mitochondria. In the presence of stress, such as ischemia-reperfusion or exposure to a hazardous substance, additional superoxide (O_2_*-) is produced, resulting in the formation of secondary reactive species such as hydrogen peroxide (H_2_O_2_) and peroxynitrite (ONOO). These oxidants not only damage regions of the mitochondria but also alter calcium signaling, which initiates a chain reaction that results in cell death [15]. The above trends are evidenced by experimental evidence of renal cell models treated with nephrotoxicants like 1,2-dichlorovinyl-L-cysteine (DCVC), which have shown a strong correlation among the increased intracellular levels of Ca²⁺, degraded DPsm levels, and increased hydroperoxide production. Diphenylphenylenediamine and deferoxamine are antioxidants that have been shown to reduce these effects, highlighting the importance of the interplay between calcium dysregulation, oxidative stress, and mitochondrial integrity [14]. Overall, there is a strong interconnection between the capacity of mitochondria to maintain redox homeostasis and calcium buffering. Not only does their perturbation prevent energy consumption, but it also increases the sensitivity of cells to apoptotic signals-mechanisms that are closely connected to the pathogenesis of neurodegenerative diseases [15].

### 1.3 Glial Cells and Their Interactions with Mitochondrial Function

The central nervous system (CNS) is made up of neurons and an extensive number of glial cells, which outnumber neurons and are needed to play important supportive, regulatory, and protective functions [16]. The major glial cells are astrocytes, oligodendrocytes, and microglia. They must stabilize the brain, respond to injury, and build neuron cycles. Recycling of neurotransmitters, maintenance of the blood-brain barrier, and provision of neurons with metabolic support require the presence of astrocytes. They also assist in the maintenance of appropriate levels of ions outside of cells and provide neurons with lactate when it is most needed. Astrocytic mitochondria, particularly those adjacent to the synapses, are highly important in buffering neuronal Ca²⁺ and reducing ROS [17]. The myelination of CNS axons is carried out by oligodendrocytes, and it accelerates the transmission of signals. Not only do these glial cells provide the axons with metabolic substrates such as pyruvate and lactate to sustain them and provide sufficient energy, but they also sheath the axons structurally. Microglia, the brain's immune cells, constantly monitor their surroundings and react to any harmful stimuli. Microglia transform their metabolism when activated, implying an increased glycolysis activity and alterations in the mechanism of mitochondrial functioning. These changes modify their ability to induce inflammation and mitochondrial issues are likely to promote a pro-inflammatory phenotype [18]. The glial cells cooperate with the neurons as well as with one another to manage immunological reactions, energy allocation, and the remodeling of the synapses. Many neurodegenerative disorders have been linked to disruption of the mitochondrial functioning during glial cell activity (Fig. **1**). As an example, impaired mitochondrial quality maintenance in astrocytes may lead to glutamate excitotoxicity, but the microglial mitochondrial dysfunction contributes to chronic neuroinflammation. It is also important to understand that the interactions between neuronal and glial mitochondrial activity are important in the explanation of the molecular pathways of neurodegeneration [19].

### 1.4 Mitochondrial Dysfunction in Parkinson’s Disease

The malfunction of the mitochondria is an important pathogenic feature of Parkinson's disease (PD), which is a progressive neurodegenerative disease that affects mostly the dopaminergic neurones of the substantia nigra pars compacta (SNc). PD is characterized by both motor and non-motor signs and symptoms, such as bradykinesia, resting tremor, and rigidity, sleep disturbances, depression, and autonomic dysfunction [20]. Although PD is a condition with a significant impact on numerous individuals, the disease has no cure or prevention methods. One of the notable obstacles to the progress of the treatment is the poor understanding of the underlying molecular pathways. One of the most frequent cellular alterations observed in PD [21] is the accumulation of misfolded α-synuclein as Lewy bodies, which are proteinaceous inclusions within the susceptible neuron groups. These Lewy bodies are patchy and mostly in areas of the brain with active mitochondria. The α-synuclein aggregation is found to disrupt the functioning of the mitochondria through interference with the electron transport chain, alteration of mitochondrial dynamics, and weakening of the mitochondrial membrane potential. In addition, it has been shown that α-synuclein can propagate trans-synaptically using animal models, and this finding supports the idea that the pathology of Parkinson's disease is spread by a prion-like mechanism [21].

Also, other family-related genes of Parkinson's disease that belong to the α-synuclein group include PINK1, PRKN, and DJ-1, which encode proteins that are directly involved in ensuring the maintenance of the integrity of the mitochondrion. These genes mutate, leading to defective mitophagy, increased reactive oxygen species (ROS) production, and increased susceptibility to oxidative damage [22]. Mitochondrial failure in such a case is usually accompanied by neuroinflammation, and thus, there is a two-way interaction of mitochondrial stress leading to immunological responses and inflammation exacerbating the damage to the mitochondria. Recent findings highlight the interplay of complexes between mitochondrial health and neuroinflammatory signalling. Using PD models as an example, mitophagy that fails to do its job results in the release of mitochondrial DNA and other types of damage-associated molecular patterns (DAMPs) that activate innate immune receptors such as the cGAS-STING pathway and lead to increased production of inflammatory cytokines such as IL-6. This cascade accelerates the loss of neurons and supports the concept that mitochondrial failure is a cause and also an effect of disease progression in PD. Thus, a combination of mitochondrial failure, α-synuclein pathology, and neuroinflammation indicates that PD has a complex network of diseases (Fig. **2**). The health of mitochondria is a promising approach to curing diseases, and it may be possible to alter the course of the disease and improve its clinical outcomes [23].

## 2 MITOCHONDRIAL BIOENERGETICS IN NEURONS AND GLIAL CELLS

These electrons pass through a series of enzyme complexes, producing a proton gradient across the inner mitochondrial membrane. This gradient drives ATP synthase to convert ADP and inorganic phosphate into ATP. This process, known as oxidative phosphorylation, is highly efficient [24]. The role of mitochondria extends beyond energy production; they are also involved in proteostasis, lipid synthesis, ROS regulation, and immunological signaling [25].

Mitochondria play a critical role in the cellular functions; however, their impairment is largely related to the etiology of neurodegenerative diseases, particularly Parkinson's disease. Mitochondrial dysfunction is one of the major pathogenic processes in Parkinson's disease [26]. It comprises ETC dysfunction, especially at Complex I (CI), which produces less ATP and more reactive oxygen species (ROS). Genetic defects in genes like PINK1, Parkin, and α-synuclein involved in PD disrupt mitophagy, which causes accumulation of damaged mitochondria and increases oxidative stress.

Before using animal models, it is important to note that Reference [27] has shown that mitochondria-targeting poisons, such as MPTP and rotenone, can directly inhibit complex I (CI), thereby recapitulating the mitochondrial impairment seen in Parkinson's disease and demonstrating that mitochondria are a key target in the disease's pathogenesis. MPTP and rotenone are also common neurotoxins that inhibit the activity of mitochondrial complex I (orange blunt arrow). This causes oxidative phosphorylation to stop, ROS to increase, and dopaminergic neurons to die, all of which are symptoms of PD. Complex I (red blunt arrow) can be hindered by any mutations in mitochondrial DNA (mtDNA) that can be inherited or acquired during life, which worsens the energy failure and promotes the development of the PD-like features (Fig. **3**). The interaction of all these genetic and environmental factors causes an increase in the level of ROS, an increase in mitochondrial calcium retention, and the opening of the mitochondrial permeability transition pore (mPTP) [25]. This cascade prevents the formation of ATP and accelerates the death of dopaminergic neurons. To recreate this pathology, neurotoxins such as MPTP and rotenone, which act in a direct manner on mitochondria, are used to study models of Parkinson's disease. These models are crucial in that they offer significant evidence to show that mitochondrial dysfunction is not just a mere result but a trigger of Parkinsonian neurodegeneration [26].

### 2.1 Mitochondrial ATP Production (Tricarboxylic Acid Cycle)

The neurons and glial cells produce ATP primarily in mitochondria. The predominant metabolic pathway that encourages oxidative phosphorylation is the TCA cycle. Mitochondria, particularly neurons and glial cells, synthesize the majority of adenosine triphosphate (ATP) required by the brain to function well [27]. Neurons are highly active in synaptic activity, and glial cells are essential for maintaining homeostasis by providing ATP; both cell types require mitochondria. The Krebs cycle, or TCA cycle, converts acetyl-CoA, which is formed as a result of glucose, fatty acids, and amino acids, into NADH and FADH_2_. This is what powers the ETC. These enzymes provide electrons to the ETC, which leads to the movement of protons across the inner membrane of the mitochondrion. This creates a gradient that makes ATP synthase turn ADP and inorganic phosphate into ATP. Oxidative phosphorylation is a very effective way to do this [28].

Mitochondria are important for cell health because they control apoptosis, calcium balance, macromolecule biosynthesis, and inflammation, in addition to producing ATP [14]. The TCA cycle is a major part of metabolism. The cycle starts with the condensation of acetyl-CoA and oxaloacetate to make citrate. Citrate is then changed into isocitrate, α-ketoglutarate (α-KG), succinyl-CoA, succinate, fumarate, malate, and ultimately oxaloacetate [29].

#### 2.1.1 Steps that Give off Energy are

Oxidative decarboxylation (which makes NADH), substrate-level phosphorylation (which makes GTP → ATP), and FAD reduction to FADH_2_ (*via* succinate dehydrogenase, which is also a part of Complex II in the ETC). Thereby, neuronal and glial energy metabolism relies on a working TCA cycle and mitochondrial oxidative phosphorylation.

### 2.2 Mechanism of Metabolic Substrate Utilization

#### 2.2.1 Insulin Resistance

Research indicates that around 58.4% of individuals with Parkinson's disease show insulin resistance [29], a significant metabolic change that connects diabetes with Parkinson's disease (PD). In this condition, disease characteristics deteriorate, progression accelerates, and the probability of acquiring Parkinson’s disease dementia (PDD) escalates [30]. Insulin receptor expression is diminished in critical brain areas, including the substantia nigra (SN) and basal ganglia. Chronic hyperinsulinemia-elevated insulin in both blood and brain-alters insulin transport, contributing to dopaminergic neuronal loss [31]. Furthermore, newly diagnosed PD patients have reported elevated levels of insulin-like growth factor-1 (IGF-1) caused by microglial activation, suggesting early degenerative and inflammatory alterations.

#### 2.2.2 Hyperglycemia

Hyperglycemia causes impaired neuronal signaling, especially in brain regions governing motor control and dopamine regulation. It promotes neuroinflammation, impairs autophagy, disrupts mitochondrial function, and interferes with dopamine metabolism. Methylglyoxal, a toxic glycolytic byproduct, accumulates during hyperglycemia and intensifies oxidative stress by damaging mitochondria and dopaminergic neurons. Given the high energy demands of SNc dopaminergic neurons, this leads to extreme vulnerability to damage [31]. Neuroinflammation, mitochondrial dysfunction, impaired protein degradation due to lysosomal dysfunction, and dopamine oxidation and quinone production related to VMAT2 reduction are all indirect effects of hyperglycemia that lead to oxidative stress in dopaminergic neurons. Oxidative stress is further amplified by hyperglycemia-induced activation of oxidative stress transcription factors (Nrf2 and FOXO1), reduction in catalase, and elevation of TXNIP (a thioredoxin inhibitor) in caudate putamen. The combined action makes the mitochondria more permeable and causes apoptosis, which stops the clearance of α-synuclein and starts its aggregation. The interaction of BBB failure, oxidative stress, insulin resistance, and glycated alterations engenders a complex pathogenic milieu that contributes to PD [32].

#### 2.2.3 Oxidative Stress

The resulting oxidative stress is caused by impaired glucose metabolism and energy deficits, especially in SNc dopaminergic neurons, which are more susceptible to it owing to their high oxidative load and dopamine metabolism. The stress factors in PD are mitochondrial problems, insulin resistance, and excessive blood sugar. Hyperglycemia disrupts the redox balance by increasing the reactive oxygen species (ROS), inhibiting antioxidant processes, and promoting mitochondrial damage. It also reduces the activity of glucocerebrosidase that causes lysosomal dysfunction and accumulation of α-synuclein [33]. During the oxidation of dopamine, it forms semiquinone and quinone, which damage neurons, increase ROS, and increase the permeability of mitochondria. These processes prevent neurotransmission and result in the death of dopaminergic cells, which trigger both motor and non-motor symptoms of Parkinsonian disease.

#### 2.2.4 Glycated Alteration

Methylglyoxal, which forms when the blood sugar is excessively glycosylated, binds to α-synuclein, making it oligomerize and aggregate and become insoluble. This prevents the ubiquitin-proteasome system, the autophagy-lysosome pathway, and the heat shock protein responses from clearing a-synuclein. The affinity of glycated α-synuclein to vesicular membranes is lower, and this influences vesicular trafficking and synaptic activity. These accumulating harmful protein aggregates kill dopaminergic cells, increase the amount of ROS, and complicate the functioning of neurotransmitters, all of which significantly deteriorate motor issues as well as cognitive dysfunction in PD [34].

#### 2.2.5 BBB Failure

In patients with PD, the BBB is also not functioning properly, as indicated by erythrocyte leakage, hemosiderin deposits, and degradation of tight junctions. In PD patients, especially those with diabetes, the increased CSF/plasma albumin ratio indicates a disruption of the blood-brain barrier. Insulin resistance causes pericyte degradation, microglia-vessel misregulation, and endothelial dysfunction, which weakens the BBB. Hyperglycemia exacerbates this by promoting oxidative stress and inflammation. As a result, α-synuclein may cross from erythrocytes to the brain, intensifying neuroinflammation [35]. BBB breach also facilitates microglial activation, lipid peroxidation, and ferroptosis in the SNpc, accelerating PD progression (Fig. **4**).

### 2.3 Oxidative Phosphorylation

The release of dopamine, a neurotransmitter vital to neuronal function, occurs in the basal ganglia *via* the substantia nigra pars compacta (SNpc). Mitochondria produce around 90% of the energy neurons need, with OxPhos being the primary pathway. Reduced ATP synthesis in neurons causes death when complex I, a critical molecule in SNpc, is deficient. Postural instability, bradykinesia, stiffness, and resting tremors are all signs of Parkinson's disease. A lack of smell, mental fogginess, insomnia, sadness, and lethargy are all examples of nonmotor symptoms. Lewy bodies, which are aggregates of alpha-synuclein, cause the loss of dopamine and dopaminergic neurons from SNpc [36]. Metabolism studies in Parkinson's disease are fascinating since SNpc has an immediate supply of ATP. The oxidative phosphorylation pathway generates membrane potential *via* proton translocation and is responsible for the majority of mitochondrial ATPs. Insufficient complex I activity due to PD-associated gene mutations may compromise mitochondrial homeostasis and kill cells. PD gene anomalies may affect ATP, Ca^2+^ ions, complex I activity, dopamine metabolism, fission-fusion dynamics, membrane potential, PTP, and ROS [37].

### 2.4 Glial Cells and Energy Metabolism

Adapting quickly to changes in the brain microenvironment and modulating their roles, glial cells are very versatile in terms of their metabolic process [38]. There are various methods of ATP production in these cells, and they are able to utilize a great variety of metabolic precursors, including lipids, carbs, and proteins. The glial cells can quickly change their functions in response to the changing microenvironment in the brain because of their metabolic flexibility. The SNpc has fewer astrocytes than the other regions of the brain, and this makes them more likely to experience oxidative stress and energy failure [39]. Astrocytes release neurotrophic factors, including metabolic astrocyte-derived neurotrophic factor (MANF), brain-derived neurotrophic factor (BDNF), and glial cell line-derived neurotrophic factor (GDNF). These factors help manage neuronal bioenergetics and keep dopaminergic neurons alive. Astrocytomas in PD brains are reactive and have a role in neuroinflammation. Fig. (**1**) shows that the SNpc of older people has larger densities of microglial cells than other parts of the brain. Additionally, there are more activated microglia and pro-inflammatory cytokines in this area [34]. Genes that are associated with Parkinson's disease (PD), such as PARK7, PINK1, and LRRK2, regulate glial cell inflammation, oxidative stress, and dysfunction of mitochondria. Infiltrating cells, such as neutrophils and T-lymphocytes, may exacerbate inflammation in neurodegenerative diseases like PD [39].

## 3 FEATURES OF GLIAL MITOCHONDRIA

An intricate network of neurons, macroglia, and microglia makes up the central nervous system (CNS). The CNS relies on macroglia and microglia, whereas neurons transmit, process, and store information. There is substantial regional heterogeneity within the glial population, with 19-40% belonging to the astrocyte subset, which originates in the neuroectoderm [40]. Post-differentiation gene expression determines astrocyte morphology, function, and molecular identity. They interact with other glia, neurons, and capillary endothelial cells *via* numerous radial pathways [41]. Gap junctions allow astrocytes and oligodendrocytes to communicate within the brain, forming a vast network. The energy generation and death of neuronal cells are connected to the metabolism of mitochondria in the brain. While neuronal mitochondria have been extensively investigated, the function of glial mitochondria, especially in pathological contexts such as Parkinson’s disease (PD), is still little examined. Mitochondria in glial cells help with calcium buffering, redox regulation, and biosynthetic support. Astrocytes mainly make ATP through glycolysis. They use mitochondria for signaling and regulatory purposes. In a healthy brain, glial mitochondria help keep the balance of antioxidants, calcium levels, and redox levels. In Parkinson's disease, astrocytic and microglial mitochondria exhibit diminished membrane potential, compromised oxidative phosphorylation (OXPHOS), and increased reactive oxygen species (ROS) levels, alongside deficient mitophagy. These modifications make it harder for glial cells to nourish neurons, make inflammation last longer, and make neuronal degeneration worse [42]. Moreover, mutations associated with Parkinson's disease in genes including PINK1, Parkin, and DJ-1 interfere with mitochondrial quality control, dynamics, and autophagy in glial cells, exacerbating neurodegenerative processes.

### 3.1 Astrocyte and its Regulation

The brain only makes up 2% of the body's weight, yet it consumes 20% of its energy for oxidative metabolism. Synapses use the majority of this oxidative energy to maintain stable ionic gradients and to absorb and recycle neurotransmitters. On the other hand, astrocyte metabolic processes use 5-15% of the brain's ATP [43]. Glucose is still the major source of energy, although lactate is a useful backup when there is a lot of demand. Astrocytes are naturally glycolytic, as shown in Cox10 mutant mice, where lactate generation makes up for less mitochondrial respiration. Even so, they are still crucial for supporting neurons. In Parkinson's disease, dysfunctional astrocytic mitochondrial activity results in diminished secretion of neurotrophic factors such as GDNF and BDNF, disrupted synaptic homeostasis, and decreased neuroprotective efficacy [43]. Other energy sources can power the adult brain, but glucose is its main energy source. Transport of lactate *via* the BBB supplies 8-10% of the energy the brain needs under restful circumstances and 20-25% during energy-intensive activities. When plasma lactate levels rise, CNS lactate absorption rises, and glucose uptake decreases. Astrocytes are essential to neuron metabolism. Astrocytes are glycolytic because Cox10 mutants produce more lactate, showing that they can meet their energy needs without OXPHOS (Fig. **5**). While astrocytes prefer glucose over lactate as an energy source, glucose absorption does not match anticipated bioenergetic output [44]. Fructose-6P is generated when glucose goes through phosphorylation inside cells, first as glucose-6-phosphate (P). The glucose-6-phosphate route is involved in glycogen production or the pentose phosphate pathway. One-carbon units are transferred *via* folate intermediates by serine and glycine for purine and thymidylate synthesis, NADPH generation, and methylation processes.

### 3.2 Lactate Metabolism and Astrocytic Support in PD

Astrocytes convert glucose to lactate *via* glycolysis and shuttle it to neurons through the astrocyte-neuron lactate shuttle (ANLS), supporting neuronal oxidative metabolism. This transfer depends on monocarboxylate transporters (MCT1/4 in astrocytes and MCT2 in neurons). In PD, α-synuclein aggregates impair MCT1/4 expression and function, reducing lactate export and impairing the neuronal energy supply. Additionally, decreased activity of lactate dehydrogenase (LDH5) in astrocytes and LDH1 in neurons disrupts the lactate-pyruvate balance, impairing mitochondrial respiration in neurons [45]. Under oxidative stress conditions typical in PD, astrocytes exhibit increased glycolysis but fail to provide sufficient lactate to neurons due to compromised mitochondrial health and altered redox status, thereby exacerbating neuronal energy crisis and degeneration [46]. In contrast to neurons, astrocytes have a reduced oxidative metabolism and an enhanced glycolytic rate; they additionally utilize more glucose. Astrocytes secrete lactate into the extracellular environment if a large quantity of glucose enters the glycolytic pathway [46]. The glycolytic nature of astrocytes, along with their preference for generating lactate instead of allowing pyruvate to enter the TCA cycle, is driven by a gene expression profile that includes various transporters and enzymes. Glycolysis is enhanced by fructose-2,6-P2 allosteric activation of PFK in astrocytes caused by elevated Pfkfb3 expression. Astrocytes' mitochondria have very low levels of aspartate/glutamate carrier (AGC), a protein involved in the malate-aspartate shuttle, which brings reducing equivalents (NADH) from the cytosol to the mitochondria. Here, the cytosolic conversion of glycolytically generated pyruvate to lactate keeps the NAD+/NADH ratio high, which is required to keep the glycolytic rate high. Lastly, astrocytes may be more likely to convert pyruvate to lactate if pyruvate dehydrogenase, an important enzyme controlling pyruvate's entry into the TCA cycle, is inactivated due to phosphorylation or has low expression levels [47].

### 3.3 Anaplerosis in Astrocyte Mitochondria and Its Impairment in PD

Astrocytes use anaplerosis to make oxaloacetate from pyruvate carboxylase (PC), which is how they acquire TCA cycle intermediates back. This helps with biosynthesis and controlling neurotransmitters. In Parkinson's disease, stress on mitochondria lowers the expression and activity of PC, which lowers the levels of TCA intermediates and makes it harder to make neurotransmitter precursors like glutamate and GABA. This metabolic deficiency undermines neuron-glia connections and exacerbates synaptic dysfunction. Additionally, alterations in mitochondrial transporters associated with Parkinson's disease and diminished pyruvate influx into mitochondria exacerbate the inefficiency of anaplerotic processes, resulting in a loop of metabolic failure [48]. FAs/ketone bodies, glutamine/glutamate, and pyruvate drive mitochondrial oxidative metabolism. Upon entering the matrix of mitochondria by means of the mitochondrial pyruvate carrier (MPC1), PDH converts pyruvate to acetyl-CoA. This process is critical for preserving oxidative carbon flow when energy is being used. The TCA cycle produces intermediates that may be used in the biosynthesis of many substances. One anaplerotic enzyme in mitochondria, called pyruvate carboxylase (PC), converts pyruvate into oxaloacetate (OAA) to replenish carbons that are lost in the TCA cycle *via* citrate, OAA, and αKG export. The brain consumes 3-10% of glucose *via* PC for energy generation, while astrocytes use 33-50% of pyruvate approaching mitochondria during anaplerotic activities. It has been shown that PC has a mitochondrial pyruvate metabolism that is 4.3 times greater than PDH [49]. In astrocyte gluconeogenesis, PEP carboxykinase converts OAA to PEP in the mitochondria. Although PEP may be shown to leave mitochondria, the exact process by which it does so remains unknown. Astrocytes express glucose 6-phosphatase and fructose 1,6-biphosphatase (FBP), making gluconeogenesis brain-specific. Alanine, aspartate, glutamate, and lactate are precursors for astrocyte gluconeogenesis, which is becoming a more essential glucose source for neurons under stress [50]. Astrocytomas, microglia, oligodendrocytes, and ependymal cells are the main cell types that express PC. The phosphorylation of PDH in astrocytes decreases PDH activity, implying that the TCA cycle requires a carbon source other than Ac-CoA. The physiological consequences of fatty acid oxidation (FAO), which is the preferred mechanism for producing Ac-CoA in astrocytes, remain unknown.

### 3.4 Dysregulated Pyruvate Metabolism in PD

In astrocytes, pyruvate generated from glucose is primarily converted to lactate due to active pyruvate dehydrogenase kinase (PDK), which inhibits pyruvate dehydrogenase (PDH). This maintains high glycolytic flux by preserving NAD+/NADH balance. In PD, impaired PDH activity-caused by oxidative stress, mitochondrial dysfunction, and α-synuclein accumulation-limits pyruvate oxidation, decreasing ATP production in astrocytes. Additionally, HIF-1α signaling, which regulates glucose metabolism and lactate export, becomes dysregulated in PD astrocytes. This impairs GLUT1, MCT4, and HK2 expression, thereby disrupting glucose uptake and conversion into lactate under hypoxic or inflamed conditions [51]. These disruptions further isolate neurons from their metabolic support system, accelerating degeneration. Astrocytes contain an excess of pyruvate, which is phosphorylated by PDK, because of their high glucose consumption; this excess restricts the amount of pyruvate that may enter the TCA or Krebs cycle. In contrast to astrocytes, neurons regulate glycolysis by breaking down PFKFB3, an enzyme that produces fructose-2,6-P, an allosteric activator of PFK 1. In order to recycle NADPH for reductive GSH processing, neurons that are under oxidative stress redirect their glucose utilization to PPP. A large amount of glucose is used by astrocytes to generate lactate, which is then transported to neurons for OXPHOS [52]. Through endoplasmic reticulum sequestration, this shuttle travels from the periphery of blood vessels to the processes surrounding synapses. Astrocytes transport lactate to neurons in hypoxic conditions by activating HIF-1α, which upregulates glycolytic enzymes such as MCT4. Through the action of HIF-1α, nitric oxide enhances GLUT1, hexokinase-2, and MCT4. According to other studies, protecting neurons from hypoxia damage might be possible by deleting HIF-1α from astrocytes. New evidence that challenges the ANLS concept suggests that neurons may increase glucose metabolism in response to energy needs or aging [53].

### 3.5 Glutamate-glutamine Cycle Dysfunction in PD

Astrocytes regulate extracellular glutamate through excitatory amino acid transporters (EAATs) and convert it into glutamine using glutamine synthetase (GS). This cycle maintains synaptic transmission and prevents excitotoxicity. In PD, mitochondrial impairment and reduced astrocytic ATP impair EAAT function and GS activity, leading to glutamate accumulation and excitotoxicity. Additionally, α-synuclein reduces the expression of MCTs and LDH5, impairing the lactate shuttle and compounding energetic stress in neurons [54]. Disruption of the glutamate-glutamine cycle leads to decreased glutamate clearance and altered neuronal excitation, contributing to motor and cognitive symptoms of PD. The release of glutamate (Glu) across the synaptic junction stimulates glutamatergic receptors (GluR), which in turn is associated with the high energy consumption of a neuronal compartment. Astrocytes use excitatory amino acid transporters (EAATs), namely GLT-1 and GLAST, and three Na^+^ ions to absorb the majority of glutamate that is released at synapses. To release Na^+^ ions, the Na^+^/K^+^ ATPase needs ATP. The expression of GLUT1 by astrocytes and capillary endothelial cells leads to the absorption of glucose from the blood and its nonoxidative utilization. Monocarboxylate transporters (MCT1 and MCT4 in astrocytes and MCT2 in neurons) carry lactate to neurons after it has been converted to pyruvate by lactate dehydrogenase 5 (LDH5, which is prevalent in astrocytes) [55]. The enzyme lactate dehydrogenase 1 (LDH1) is mostly found in neurons and converts lactate to pyruvate (Pyr), which neurons may utilize as an energy source. Neuronal glucose transporter 3 (GLUT3) is another mechanism by which neurons may absorb glucose. Through the glutamate-glutamine cycle, astrocytes also participate in the recycling of synaptic glutamate. Glutamine synthetase (GS) converts glutamate to glutamine (gln) in astrocytes, which glutaminases (GLS) convert back to glutamate in neurons [56].

## 4 CALCIUM HOMEOSTASIS AND MITOCHONDRIA IN NEURONS AND GLIA

Mitochondrial Ca^2+^ signalling is very important in the death of nigral dopaminergic neurons in Parkinson's disease (PD) because it controls ATP production and mitochondrial oxidant stress. In the substantia nigra pars compacta (SNpc), the overexpression of α-synuclein elevates cytosolic Ca^2+^, leading to increased mitochondrial calcium levels and oxidative stress. Research indicates that LTCC (L-type calcium channel)-mediated Ca^2+^ influx increases oxidative stress, resulting in dopamine oxidation, α-synuclein misfolding, and diminished lysosomal function in Parkinson's disease [57]. In Parkinson's disease, inhibiting Cav1 channels or mitochondrial Ca^2+^ uptake safeguards dopaminergic neurons from oxidative injury, hence endorsing the therapeutic promise of Ca^2+^ regulation [58]. Ca^2+^-dependent systems, including Parkin and PINK1, control mitochondrial quality control, which includes mitophagy. In Parkinson's disease models, the absence of PINK1 disrupts mitochondrial calcium efflux, resulting in calcium overload. Concurrently, LRRK2 mutations modify the expression of MCU and MICU1, diminishing the mitochondrial calcium buffering capacity [58]. Recent discoveries indicate that disruptions in mitochondrial-associated membranes (MAMs), which enable Ca^2+^ exchange between the endoplasmic reticulum and mitochondria, are altered in Parkinson's disease (PD), compromising the fragile equilibrium between Ca^2+^ signalling and mitochondrial metabolism. This imbalance leads to synapse failure and speeds up the loss of dopaminergic neurons.

### 4.1 Ca2+ Homeostasis Functions in Neurotransmitter Release in the Brain

The release of neurotransmitters is triggered by the influx of Ca^2+^ at the presynaptic terminal that is initiated by an action potential. Such a rise in Ca^2+^ leads to vesicle fusion and exocytosis in the synapse, which are largely regulated by synaptotagmin (Syt1), complexin, and other Ca^2+^ sensors [59]. Ca^2+^ influences both the synchronous and asynchronous release modalities and spontaneous neurotransmission is correlated with baseline changes of Ca^2+^. The marked changes in the buffering of Ca^2+^ by the synaptic reuptake response and the disruption of vesicle fusion machinery impairs neurotransmission facilitated by Ca^2+^ in Parkinson disease. Animals deleted in Syt1 have reduced synchronous release, yet they continue with asynchronous responses, which indicate the complexity of Ca^2+^-dependent release processes [60]. The even more complicated fact is that the interactions between SNARE proteins and the process of vesicle docking that are related to PD alter and complicate the release of neurotransmitters even further. The balance of glutamatergic and GABAergic neurotransmission may be disrupted by chronic dysregulation of Ca^2+^ in presynaptic terminals in contributing to non-motor symptoms of Parkinson's disease, such as mood disturbances and cognitive impairment.

### 4.2 Mechanism of Ca2+ Signaling and Neuronal Excitability in Neurodegenerative Diseases

Ca^2+^ signalling regulates synaptic plasticity, the ease with which neurons fire, and up-down brain rhythms. The disturbance of these systems is a cause of neurodegenerative diseases including Parkinson disease, Alzheimer disease and schizophrenia [61]. The effect of excitatory input is altered by ion channels such as Kv7.2/7.3 (controlled by PIP2), SK/BK (controlled by Ca^2+^), and HCN (controlled by norepinephrine and dopamine). In PD, motor and cognitive functions are influenced by an abnormal dopaminergic modulation in terms of Ca^2+^-dependent excitability and neurotransmission. The imbalance in the regulation of intracellular Ca^2+^ can stimulate hyperexcitability of neurons and cell death. High neuronal excitability, especially in circuitry in the basal ganglia is a characteristic of early PD and closely associated with the disrupted Cav1.3-mediated Ca^2+^ currents. The gradual degeneration of the pacemaking activity of SNc neurons is regarded as a possible early biomarker of PD development [62].

### 4.3 Ca2+ Homeostasis and Neuronal Firing Mechanisms in Normal and PD-Affected Brains

Neurons rely on closely controlled concentrations of intracellular Ca^2+^ (~100 nM), and elevations in concentrations induce neurotransmitter release and intracellular signaling [63]. Membrane potential, gene expression and neurotransmitter cycling are regulated by calcium ions (Ca^2+^) using energy-requiring pumps and exchangers. Disturbed calcium homeostasis leads to impaired synaptic transmission as well as dysfunction of mitochondria in Parkinson disease. Excess calcium in the cytosol leads to oxidative stress and apoptosis and calcium signalling to fail due to ageing or due to disease leads to the slow death of neurons. Evidence of research studies of Parkinson disease models suggests that mitochondrial calcium overload is able to not only impair synaptic plasticity, but also impair ATP production, thus greatly increasing the impairment of neuronal firing reliability. The reduced shooting precision in the Parkinson disease is directly linked to the energy burden caused by the dysregulation of calcium [64].

### 4.4 Ca2+ Homeostasis Dysfunction and Cell Death Pathways in PD

Ca^2+^ ions regulate such functions as gene expression, metabolism, and apoptosis. Mitochondria play a role in the buffering of Ca^2+^ and redox state control. Mitochondrial elimination, mitophagy, is also controlled by Ca^2+^ signals [65]. In PD, dopaminergic neurons die more quickly, and the excess of Ca^2+^ in the mitochondria is caused by the malfunction of the exchangers (*e.g*., NCLX, LETM1), which results in oxidative stress and reduced mitophagy. Intrusions in Ca^2+^ transport, including ER-mitochondria Ca^2+^ transfer and mitCU regulation, are now gaining recognition as essential elements in the pathogenesis of PD [65]. When Ca^2+^ opens the mitochondrial permeability transition pore (mPTP), it is likely a significant contributor to cell death in PD. Sustained elevation in mitochondrial Ca^2+^ promotes mPTP activation, leading to the release of cytochrome c and triggering intrinsic apoptotic cascades. Novel compounds targeting mPTP and Ca^2+^ uniporters are being investigated as neuroprotective agents in PD therapy.

## 5 MITOCHONDRIAL ROS PRODUCTION AND REDOX BALANCE

The production of ATP by the organelle known as mitochondria is one of its primary functions in the cell. Cells produce this energy by respiration, which uses mitochondrial complexes I, III, and IV of the ETC to pump protons and to synthesize ATP. A cross-linked component of mitochondrial activity, the mitochondrial membrane potential (∆ψm) maintains the shape of the organelle and serves as a proton motive force for ATP generation [66]. Depending on the energy needs, the mitochondrial distribution in cells changes. The brain uses about 10 times as much oxygen and glucose as other tissues, even though myocytes are denser than neurons. Most mitochondrial abnormalities or poisons damage brain function and neurological illness due to the brain's high energy requirement and ATP synthesis and consumption. Free radicals, mostly ROS, are produced by mitochondria owing to their high oxygen accessibility [67]. There is a strong interdependence between the rate of ROS generation, the activity of ETC complexes, and the variance in mitochondrial membrane potential (∆ψm). Uncoupling reduces ROS production, but respiration inhibition increases mitochondrial membrane potential dissipation [68]. Mitochondrial hyperpolarization may exacerbate oxidative stress. When electrons are released from the electron transport chain, and free radicals are formed, the electron transport chain (ETC) begins to produce reactive oxygen species (ROS). Superoxide radicals from electron leakage from the ETC convert to H_2_O_2_.

### 5.1 Mitochondria as a Source of Reactive Oxygen Species (ROS)

Enzymes in the ETC, mitochondrial antioxidant enzymes (MAO), and hydrogen peroxide (H_2_O_2_) are all examples of reactive oxygen species (ROS). An excess of reactive oxygen species (ROS) may accelerate lipid peroxidation and the inositol triphosphate-dependent calcium signal. For complex I, PARP may be activated when NADPH oxidase in PS or AD produces too much ROS. PARP then uses NAD and decreases NADH. Inhibition of the glucose transporter and induction of mitochondrial substrate restriction may occur as a result of mitochondrial ROS generation [69]. Effective antioxidant mechanisms keep the mitochondrial matrix in redox equilibrium. Rapid spontaneous or enzymatic dismutation to hydrogen peroxide is possible for the superoxide anion, which has a lifespan of 1 ns. To protect important peptides from oxidation by reactive oxygen species (ROS), the cell's mitochondria and the remainder of the cell are supplied with the powerful endogenous antioxidant glutathione (GSH). Enzymes such as peroxiredoxin 3, glutathione peroxidase, and catalase break down permeable H_2_O_2_, which is involved in signaling cascades [70]. After external, cellular, or intramitochondrial stimuli, mitochondria create ROS using diverse methods. Mitochondrial ROS activate the inositol trisphosphate-triggered calcium signal, phospholipase C, and lipid peroxidation processes. Hypoxia triggers an increase in reactive oxygen species (ROS) in mitochondria, which in turn activates respiration and enhances the calcium signal in astrocytes. The redox state of mitochondria allows ROS to control the absorption of calcium. Multiple diseases, including neurodegeneration and cell death, are caused by an excess of reactive oxygen species (ROS) or an imbalance in the antioxidant system.

Mitochondrial complex I dysfunction is a key clinical characteristic of Parkinson’s disease (PD), which causes electron leakage through the electron transport chain (ETC) and excessive generation of superoxide (O_2_-). This reactive oxygen species (ROS) is rapidly converted into hydrogen peroxide (H_2_O_2_) by mitochondrial superoxide dismutase (SOD2). Under normal circumstances, the redox equilibrium is preserved by antioxidant systems such as glutathione (GSH), thioredoxin, and peroxiredoxins, which neutralise the reactive oxygen species (ROS) and promote mitochondrial integrity. In Parkinson's disease, there are many conditions that work together to disturb this homeostasis. The defect states introduced by mutations in genes like PINK1, Parkin, DJ-1, and LRRK2 disrupt the quality control of mitochondrial systems, and it becomes more difficult to dispose of damaged organelles that generate reactive oxygen species. Environmental toxins like MPTP and rotenone intensify oxidative stress by further inhibiting complex I. Oxidative modification of α-synuclein facilitates its misfolding and aggregation, whereas dopamine oxidation produces reactive quinones that covalently alter proteins, disrupt proteostasis, and exacerbate oxidative stress [71]. Mitochondrial reactive oxygen species (ROS) overproduction in the process of Parkinson's disease influences the processes of calcium homeostasis by suppressing mitochondrial calcium uptake and efflux to cause calcium overload and calcium overload-induced mitochondrial permeability transition pore (mPTP). This phenomenon destabilizes membrane potential (DPsm), stops the production of ATP, and releases pro-apoptotic molecules such as cytochrome c, which activate intrinsic apoptotic functions. Constant movement to pro-oxidant condition with inadequate antioxidant defence maintains a vicious circle of mitochondrial impairment, oxidative stress, and dopaminergic neuronal degeneration. Therapeutic strategies to restore redox equilibrium in Parkinson's disease include mitochondria-targeted antioxidants (*e.g*., MitoQ, SkQ1), agents that enhance endogenous antioxidant defences *via* Nrf2 activation, and pharmaceuticals that stabilise complex I functionality. Restoring mitochondrial redox equilibrium is a viable strategy for decelerating Parkinson's disease progression by disrupting the cycle of oxidative damage and neuronal degeneration. The prevalence of neurodegenerative disorders, such as Alzheimer's disease (AD) and Parkinson's disease (PD), is growing as our populations age [72].

### 5.2 OXPHOS Dysfunction in Glia

Neurons obtain energy, maintain ionic gradients, and absorb and recycle neurotransmitters from high OXPHOS rates. While astrocytes are very glycolytic (Fig. **2**), they leak lactate into the extracellular space after converting a considerable amount of glucose. As a fuel for OXPHOS in neurons, lactate is shuttled between astrocytes and oligodendrocytes (Figs. **1** and **2**); according to the astrocyte-neuron lactate shuttle hypothesis [73]. Astrocytes phosphorylate pyruvate dehydrogenase (PDH), which takes pyruvate into the TCA or Krebs cycle (Figs. **1** and **2**). Astrocytes have the same oxidative capability as neurons but remain mitochondrial dysfunction resistant. Astrocytes may use glutamate as a fuel source for OXPHOS; glutamate is mostly converted to glutamine by glutamine synthase (GS), but it can also be metabolized *via* the TCA cycle. The TCA cycle metabolizes more glutamate when its extracellular concentration reaches synaptic transmission thresholds, but glutamine conversion declines. Note that glutamate also stimulates glycolysis [74]. Table **1** illustrates how disruptions in mitochondrial complexes correlate with glial and neuronal degeneration. Impaired GLAST/ EAAT1 function disrupts glutamate uptake, causing excitotoxicity and neuronal stress in ALS, epilepsy, and Alzheimer’s. Problems with cytochrome c oxidase (Complex IV) and Complexes I, III, and V make it harder for cells to make ATP and raise ROS levels, which can cause oxidative damage. Complex I dysfunction is associated with ALS, Parkinson's disease, and multiple sclerosis due to diminished ATP levels and elevated reactive oxygen species (ROS). Malfunctions in Complexes III and IV also make it harder for electrons to move, which makes neurodegeneration worse. When ATP synthase (Complex V) fails, it causes energy shortages that make diseases worse. These mitochondrial abnormalities jointly compromise glial support and neuronal viability in central nervous system illnesses.

### 5.3 Mitochondrial Transcription Factor A (Tfam) (Mitochondria and its Antioxidant Defenses in Neurons and Glia)

A crucial protein for mtDNA metabolism is nuclear-encoded mitochondrial transcription factor A (TFAM). TFAM binds mtDNA sequence-specifically and non-specifically. Mitochondrial transcription, which may also be the replication RNA primer, requires sequence-specific binding to mtDNA promoter regions. TFAM may also compress the genome by binding to mtDNA without sequence. TFAM's effect on mtDNA copy numbers may include both binding modalities. TFAM regulates mtDNA copy number; thus, it's time to ask whether it's linked to neurodegeneration. The response may reveal TFAM-related mitochondrial pathways' neurodegeneration and neuroprotection potential [81]. Damage to cellular components may occur if antioxidant defenses fail to adequately neutralise reactive oxygen species (ROS), which are hazardous consequences of oxidative phosphorylation (OXPHOS) and are mostly produced by mitochondria, the "powerhouses" of the cell. To maintain the redox balance and prevent oxidative stress, neurons and glial cells in the brain, especially astrocytes, depend on a strong system of antioxidants such as glutathione (GSH), thioredoxin (Trx), peroxiredoxin (Prx), and catalase. However, mitochondrial malfunction may cause neurodegeneration since neurons have a high metabolic demand and a limited antioxidant capacity. TFAM and mtDNA are linked to neurodegenerative diseases, *e.g*., “Parkinson’s disease”, “Alzheimer’s disease," “Huntington’s disease," “amyotrophic lateral sclerosis," “aging”, and “neurodegeneration. TFAM and mtDNA play a major role in the risk of neurodegenerative diseases, cause changes in mtDNA or TFAM levels in neurodegenerative conditions, and act as a key regulator in neurodegeneration [82].

## 6 MITOCHONDRIAL DYNAMICS

Keeping mitochondrial function healthy is an important method to defend against neurodegenerative illnesses, since we now understand the connection between oxidative stress, neurodegeneration, and mitochondrial malfunction. Chronic microglial activation contributes to neurodegenerative illnesses by causing neuroinflammation, neuronal mitochondrial damage, and excessive ROS generation under pathological settings. Restoration of mitochondrial function, reduction of oxidative stress, and protection against neurodegeneration might be achieved by therapeutic techniques that target Tfam regulation and enhance mitochondrial antioxidant mechanisms. Researchers are striving to discover new therapeutic approaches to reduce neuronal damage induced by oxidative stress by focusing on mitochondrial antioxidant systems and controlling mitochondrial dynamics [83].

### 6.1 Mitochondrial Fission and Fusion in Parkinson’s Disease and Other Disorders

In Parkinson's disease, mitochondrial fission and fusion are significantly disrupted, with research indicating an imbalance towards excessive fission, resulting in fragmented mitochondria and contributing to disease progression by impairing mitochondrial function and promoting neuronal death. This perturbation is particularly related to mutations in the genes that regulate key proteins that regulate these pathways, such as Parkin and PINK1 [84]. The Parkin/PINK1 pathway tends to be disturbed in PD, resulting in excess fission and fragmentation of the mitochondria. This impairs mitochondria, increases oxidative stress, and disrupts calcium, aggravating neurodegeneration. Therapeutically managing this imbalance has the potential to restore the mitochondrial dynamics [85]. PD-related PARK genes, such as PINK1 (PARK6) and Parkin (PARK2), were studied, and their role in mitochondrial quality regulation has been clarified. Autosomal recessive early-onset PD cases are typically caused by these mutations that alter the shape of mitochondria and increase the production of more ROS. The neurons of affected individuals show malfunctioning of the mitochondria and low efficiency in breathing [86].

PINK1 and Parkin regulate fission and fusion by transmitting signals. They also alter the process of mitophagy, biogenesis, and vesicle formation of mitochondria. As they cease their production, the electron transport chain fails to function, the level of ATP decreases, and oxidative damage occurs. There is Drp1 in Drosophila that promotes fission, and Mfn and Opa1 promote fusion. Pink1/parkin mutant flies have regained mitochondrial activity with either Drp1 overexpression or Mfn mutation, which points to a conserved regulatory process [87]. GTPases such as Drp1, Mfn, and Opa1 are also proteins that assist cells in fusing and separating. These proteins break down and lose the ability to produce energy through oxidative damage when they malfunction. These changes are not peculiar to Parkinson's disease (PD) alone, but also to Alzheimer's disease (AD), where the increased fission is related to the Tau pathology and mitophagy impairment [87]. Anomaly of fusion and fission in ALS causes dead mitochondria, ATP deficiency, and increases the production of ROS. Drp1 is not functioning effectively in ALS, but the disease is frequently characterized by overactivity of Drp1 and poor functioning of Mfn1/2 and OPA1 [88].

### 6.2 Mitochondrial Mitophagy/Autophagy in Parkinson’s Disease

Selective autophagy, which is called mitophagy, removes defective mitochondria. In PD, the damage of mitophagy, which is mainly mediated by the PINK1/Parkin pathway, leads to the accumulation of dysfunctional mitochondria, a factor that promotes neuronal death [89]. PINK1 usually accumulates on damaged mitochondria and recruits Parkin, which initiates mitophagy by ubiquitinating outer mitochondrial membrane proteins. In PD, defects in PINK1 or Parkin impair this process, resulting in mitochondrial retention and an elevation in the levels of ROS [83-85]. Other suspected proteins include a-synuclein, which interacts with Miro proteins and retards the elimination of mitochondria. The recruitment of OPTN by RAB10 and the equilibrium between redox and lysosome clearance are modulated by mutations in LRRK2, DJ-1 and GBA1 [86, 87]. These findings demonstrate the complexity of mitophagy in Parkinson's disease and show that there are many treatment variations to improve the quality control of mitochondria. Mutations in PINK1, Parkin, and LRRK2 cause alterations in mitophagy pathways of individuals with Parkinson's disease (Table **2**).

## 7 INFLAMMATION-DRIVEN MITOCHONDRIAL DYSFUNCTION IN PARKINSON’S DISEASE (PD)

Parkinson's disease (PD) refers to a long-term neurodegenerative disorder that is characterized by the destruction of dopaminergic neurons of the substantia nigra. Two of the most significant modes of action of pathogens are inflammation and mitochondrial dysfunction. All these mechanisms are tightly linked together; in response to inflammation, mitochondrial dynamics, particularly fusion and fission, are disrupted, initiating cell death pathways mediated by mitochondria. Among the primary aspects that fail in PD is that alpha-synuclein (a-syn) folds improperly and aggregates, which leads to neuroinflammation and the stress of mitochondria [95]. This is a self-perpetuating process: defective mitochondria release signals that promote the further stimulation of inflammatory processes, accelerating the process of neuronal death. It has been shown through a number of studies that people with Parkinson's disease have elevated levels of inflammatory markers both in the central nervous system and the periphery, and that neuroinflammation goes beyond the brain [96]. Imaging analyses additionally correlate neuroinflammation with cognitive and visuospatial dysfunctions in patients with Parkinson's disease. Also, animal models of Parkinson's disease, including those created by 6-OHDA or a-synuclein fibrils, have confirmed that the pathogenesis of the disease is affected by the modulation of inflammation. On the molecular level, the immune cells of the brain, the microglia transform into pro-inflammatory M1 cells in PD. This property relates to the stimulation of the NLRP3 inflammasome, which is an important intracellular signalling complex that identifies stress and damage. The engagement of mitochondrial-derived signals, such as ROS, mtDNA, reduced levels of lower cardiolipin, and altered levels of mitofusin as danger-associated molecular patterns (DAMPs) initiates inflammatory cascades when activated [97-99]. These mitochondrial stress indicators are often packed in extracellular vesicles (EVs) and released into the extracellular environment, therefore increasing the inflammatory response. Another influential factor is the hypomethylated nature of the mitochondrial DNA (mtDNA), which is equivalent to the bacterial DNA. This triggers the innate immune system which causes the pattern recognition receptors (PRRs), leading to an aggravation of neuroinflammation [100, 101]. Consequently, the vicious cycle of mitochondrial damage and immunological response contributes significantly to PD. New research also demonstrates that genetic variations in specific genes of HLA predispose individuals to PD, which leads to the conclusion that inflammation is more prone to occur in individuals genetically predisposed to it. A better understanding of how these two inflammatory and mitochondrial cascades interact can provide a powerful clue in understanding why neurons die in Parkinson's disease, and it provides future treatment options to interfere with this vicious cycle.

## 8 ALTERED GLIAL METABOLISM IN NEURON-GLIA COMMUNICATION AND NEUROLOGICAL DISORDERS

Altered glial metabolism contributes significantly to the progression of Alzheimer's disease under the condition of disrupting the relationship between neurons and glia and accelerating the process of neurodegeneration. There are also similar metabolic abnormalities in glial cells that are observed in Parkinson's disease (PD) and this implies that there may be common pathogenic mechanisms in these neurodegenerative conditions. Throughout the progression of Alzheimer's disease, microglia and astrocytes experience significant changes in metabolism, rendering them ineffective in protective roles that play an important role in preserving synaptic homeostasis. Microglia which are in charge of the elimination of the amyloid-beta plaques, go into dysfunctional metabolic states decreasing their capacity to clear the protein aggregates hence exacerbating the synaptic loss [101]. Mitochondrial and glutamate mishandling occur in the astrocytes, causing excitotoxicity and neuronal damage [102]. Astrocytes in the case of Alzheimer's disease undergo astrogliosis, hypertrophy, and increased levels of glial fibrillary acidic protein (GFAP), which further increases the release of pro-inflammatory cytokines and inflames the neural system [103]. The disrupted lipid metabolism and cholesterol dysregulation lead to impairment of the ability of the glial cells to support neurons. These metabolic issues are aggravated by pathological changes, including the deposition of amyloid-beta and hyperphosphorylation of tau, and cause difficulties in neurons and glia communicating with each other. These changes would be very important to comprehend in order to formulate therapies against glial metabolism. The imbalanced glucose metabolism of astrocytes disrupts the neurotransmitter and energy balance, whereas pro-inflammatory microglia increase their dependence on glycolysis, leading to an increase in oxidative stress [104]. These metabolic disorders impair lipid metabolism, membrane integrity and brain plasticity, therefore increasing neurodegeneration. The mobilization of microglia in Parkinson's disease and the resulting alteration of metabolic pathways to pro-inflammatory ones aggravate long-term neuroinflammation and lead to the death of dopaminergic neurons. Astrocytic dysfunction, marked by diminished glutamate uptake and disrupted mitochondrial energy production, intensifies oxidative stress in the substantia nigra, hence heightening neuronal vulnerability. In addition, there is a metabolic abnormality in lipid metabolism and mitochondrial respiration within glial cells, which prevents the clearance of a-synuclein, encouraging its aggregation and additional disruption of neuron-glia communication [105]. By focusing on the similar metabolic responses in Alzheimer's disease and Parkinson's disease, it is possible to develop treatment approaches that will reduce neurodegeneration in other neurodegenerative diseases.

## 9 THERAPEUTIC APPROACHES TARGETING MITOCHONDRIAL DYSFUNCTION

### 9.1 Mitochondrial Antioxidants in Parkinson’s Disease

Mitochondria play a critical role in the metabolism of neural energy and are the main sites of the production of reactive oxygen species (ROS) during oxidative phosphorylation. Defective electron transport especially at Complex I in Parkinson's disease (PD) elevates the generation of reactive oxygen species (ROS), which leads to oxidative stress and results in the death of dopaminergic neurons. The mitochondrial antioxidants are aimed at ensuring that this oxidative stress is removed, restoring the redox balance and making the mitochondria more efficient. In the substantia nigra, which is prone to the pathogenesis of Parkinson's disease, reactive oxygen species (ROS) in the form of superoxide anions (O_2_-), hydrogen peroxide (H_2_O_2_) and hydroxyl radicals (OH) are especially dangerous. Prolonged rise in levels of ROS may destroy mitochondrial DNA (mtDNA), initiate lipid peroxidation, result in improper protein folding, and trigger apoptotic pathways. All these occur in the brains of PD individuals [106]. Therefore, the usage of mitochondrial antioxidants as a tool to cope with oxidative stress is a rational approach to treating mitochondrial dysfunction in Parkinson's disease and slowing down the progression of the disease.

### 9.2 Endogenous Mitochondrial Antioxidants and Their Role in PD

There are intrinsic antioxidant processes that help to shield the neurons from oxidative damage. In Parkinson's disease, however, these systems receive excessive or cease operation resulting in the death of neurons. The SOD family is extremely crucial in the elimination of superoxide radicals in the mitochondria. They achieve this by converting them into H_2_O_2_ which is later degraded into water and oxygen by catalase [106]. In Parkinson's disease (PD), this detoxification process is impaired, which manifests in the impaired superoxide dismutase (SOD) activity of the impacted areas of the brain. The glutathione (GSH) system also plays a very important role in maintaining redox homeostasis in dopaminergic neurons. Oxidative stress burden has been depicted with consistent reports of reduced levels of glutathione in the substantia nigra in the patient of the Parkinson's disease. The glutathione reductase and glutathione peroxidase enzymes collaborate to convert the oxidised glutathione (GSSG) into the reduced one (GSH) to maintain healthy mitochondria [107]. Thioredoxin and peroxiredoxin systems also assist in mitochondria in avoiding oxidative injury and when they fail to execute their functions correctly in PD, oxidative injury accumulates. Since oxidative stress is one of the symptoms of PD development, it is highly important to help these natural mechanisms of oxidative stress mitigation during the treatment. Increasing the activity or expression of these enzymes might be used to repair mitochondrial defects and safeguard the vulnerable dopaminergic neurons, which can alter the progression of the disease in PD.

### 9.3 Exogenous Mitochondrial Antioxidants in PD Therapy

Exogenous mitochondrial antioxidants have also been discovered as possible treatment options in order to maintain mitochondrial integrity given the high levels of oxidative stress that are involved in the development of Parkinson's disease. These chemicals either mimic or amplify endogenous antioxidant pathways to alleviate cellular damage caused by ROS particularly in dopaminergic neurons of the substantia nigra. Coenzyme Q10 (CoQ10) is a lipid-soluble antioxidant, and it is significant in the electron transport chain. The clinical research has shown that CoQ10 intake may enhance the respiration within mitochondria and decrease the oxidative injury in the Parkinson's disease models [108]. Nonetheless, experimental outcomes at a large scale indicate that we should do a better job in the administration of drugs. MitoQ, which is a CoQ10 derivative and targets mitochondria, accumulates in mitochondria and neutralises ROS directly. MitoQ has been found to reduce neuroinflammation and prevent the death of dopaminergic neurons in models of Parkinson's disease due to preclinical studies [109, 110]. According to its antioxidant properties, alpha-lipoic acid (ALA) is an antioxidant that has the ability to cross the blood-brain barrier and enhances the efficacy of other antioxidants, such as GSH, vitamin C, and vitamin E. ALA preserves the neurons of PD by stabilizing the mitochondrial membranes and preventing apoptosis by ROS [111]. Resveratrol is a polyphenol that stimulates SIRT1 signalling, promotes the proliferation of mitochondria, and prevents cellular injury by free radicals. Its role in the disease of Parkinson is associated with the improvement of the mitochondrial dynamics and reduction of the a-synuclein aggregation [112]. These chemicals collectively support the supposition that mitochondrial oxidative stress can be addressed with exogenous antioxidants and thereby prevent neuronal death and slow down the occurrence of Parkinson's disease. Table **3** is a summary of the primary biological pathways in which mitochondrial antioxidants act and their outcomes, and reveals how significant they are in terms of cell protection and disease prevention.

### 9.4 Enhancing Mitophagy in Parkinson’s Disease

A particular form of autophagy is called mitophagy, which removes damaged mitochondria to ensure good-quality mitochondria. The death of neurons is also a crucial symptom of Parkinson's disease, with the key feature of mitophagy induction being the inability to eliminate defective mitochondria, which is caused by the mutations in PINK1 and Parkin [113]. PD observes an increase in mitophagy as an effective method of brain protection. The PINK1-Parkin pathway, which plays a very significant role in mitophagy regulation fails to work properly in most instances of familial and sporadic PD. Once mitochondria experience loss of membrane potential, PINK1 accumulates on the outer side of the membrane, and attracts Parkin to label damaged organelles to destroy them [114-116]. Many strategies have proven to increase mitophagy in mice with Parkinson's disease. These include food restriction, pharmaceutical interventions, and exercise, which activate pathways such as AMPK and inhibit mTOR signalling. Such therapeutic options increase the turnover of mitochondria, are cardioprotective, reducing oxidative stress, and appealing as Parkinson's disease therapeutic options [117, 118].

### 9.5 Small Molecules that Activate Mitophagy in PD

Recent developments in the study of Parkinson's disease have revealed the existence of numerous minor compounds that facilitate the process of mitophagy and offer novel treatment methods for destroying unsustainable mitochondria and maintaining eukaryotic balance. Urolithin A is a product of polyphenols in food and enhances mitophagy as the mitochondria undergo transformation and new ones develop. Urolithin A has already shown the ability to reduce a-synuclein aggregation and recover the mitochondrial activity thereof in Parkinson's disease models [119]. Spermidine is a polyamine found in nature, which triggers autophagy by inhibiting acetyltransferase and regulating AMPK. Supplementation improves mitochondrial activity and has shown neuroprotective activity on experimental models of Parkinson's disease [120]. Besides being an antioxidant, Resveratrol stimulates mitophagy *via* the SIRT1-AMPK pathway. This assists in correcting the errors of the mitochondria and preventing death of the dopaminergic neurons [121].

Nicotinamide mononucleotide (NMN) and NIC as NAD+ precursors activate SIRT1 and PGC-1a that are necessary in mitochondrial biogenesis and quality control. They are able to increase NAD+ levels, and this process is essential to facilitate mitophagy and energy metabolism in the neurons of Parkinson's disease [122]. mTOR inhibitors such as rapamycin and torin1 enhance autophagic flow, which facilitates mitophagy. These medicines reduce oxidative stress and a-synuclein pathology, which have been important in models of Parkinson's disease [123]. Actinonin and kinetin riboside (KR) control mitochondrial proteostasis and stabilise PINK1, therefore triggering mitophagy. SS-31 (elamipretide) preserves mitochondrial membranes and suppresses ROS, and serves to enhance mitophagy in neurons with PD [124]. Overall, these small compounds comprise a possible list of medicines that can be used to repair bad mitophagy and increase the quality of mitochondria in individuals with Parkinson's disease.

### 9.6 Mitochondriogenesis Pathway PINK1-Parkin is a Mechanism of Mitophagy in PD

The PINK1-Parkin pathway is a significant approach to mitophagy. It particularly plays a key role in maintaining healthy mitochondria in neurons. This signaling plays a very critical role in the pathophysiology of Parkinson's disease (PD) where mutations in PINK1 or Parkin genes impair the process of eliminating defective mitochondria causing the death of neurons and motor impairments [125, 126]. PINK1 (PTEN-induced putative kinase 1) is carried to the normal mitochondria and rapidly degraded by mitochondrial proteins when all is well. PINK1 remains on the outer mitochondrial membrane (OMM) when the mitochondria lose the membrane potential, which can be described as an indicator of damage. After that, it auto-phosphorylates and phosphorylates ubiquitin molecules on OMM proteins. This attracts the attention of Parkin, an E3 ubiquitin ligase, to arrive and becomes active. Upon activation of Parkin, ubiquitin is conjugated to several outer membrane targets, such as VDAC1, Mfn1/2 and Miro1. It causes the autophagy adaptors (such as p62, NDP52, and optineurin) to recognise the damaged mitochondria and assist them in being incorporated into LC3-decorated autophagosomes. Finally, these vesicles merge with lysosomes, and the breakdown process is complete [125]. In Parkinson's disease (PD), the inhibition of this degradation process through genetic inheritance or sporadic mutation of PINK1 or Parkin causes the presence of defective mitochondria, which triggers amplified reactions of reactive oxygen species (ROS), calcium imbalance, and apoptotic death. This pathway is an ideal target of disease-modifying treatments due to the reduction of mitophagy, particularly in the dopaminergic neurons of the substantia nigra.

### 9.7 Enhancing Mitochondrial Bioenergetics of Parkinsonism

The restoration of the mitochondrial bioenergetics plays an essential role in the treatment of Parkinson's disease because dopaminergic neurons require increased energy that will sustain axonal movement, neurotransmitter synthesis, and neuromuscular impulse generation. Impaired oxidative phosphorylation (OxPhos), which is mitochondrial dysfunction (especially the impaired functions of Complex I), is a feature of Parkinson's aetiology, frequently linked to insufficient ATP production [127]. Some of the therapeutic options that are used to enhance mitochondrial bioenergetics in Parkinson Disease include lifestyle change interventions and medication interventions: Exercise, which is facilitated by high-intensity interval training (HIIT) and aerobic exercise, enhances the activation of PGC-1a, a transcriptional coactivator, to generate new mitochondria, breathing and mobility. AMPK and SIRT1 are also activated by exercise, and they collaborate to increase fatty acid oxidation, antioxidant defence, and ETC activity [127]. Caloric restriction (CR) and ketogenic diets boost energy metabolism by increasing the levels of NAD+, enhancing mitophagy, and shifting the production of energy to the mitochondrial pathways. These eating habits reduce the generation of ROS and increase the levels of ATP. This is particularly effective in dopaminergic neurons that are easily susceptible to oxidative damage.

The administration of nicotinamide mononucleotide (NMN) and nicotinamide riboside (NR) restores the dwindling NAD+ pools in older PD models. Their management induces SIRT1-PGC-1a signalling that enhances the production of ATP, elevates mitochondrial respiration, and enables neurons to survive [128]. Coenzyme Q10 (CoQ10) is significant to the ETC's operation since it aids in the relocation of electrons and the production of ATP. Even though a large body of clinical trials did not provide definitive outcomes, CoQ10 compounds targeting mitochondria like MitoQ have shown improved bioavailability and higher neuroprotective effects in Parkinsonian mice [129]. SkQ1 is an antioxidant of synthetic nature that ensures that the mitochondrial membrane is not affected by lipid peroxidation and makes the respiratory chain more active. Resveratrol also assists the mitochondria turnover as it activates SIRT1, and these enhance both OxPhos and redox balance in the PD neurons [112, 130]. On a molecular scale, it is possible to adjust the levels of DRP1 and OPA1 to enhance mitochondrial activity and improve the ratio between fission and fusion, leading to better mitochondrial performance and increased cell robustness [129]. The combination of these treatments makes it possible to slow down the development of PD, and enhance the survival of the neurons by maintaining the energy production of the mitochondria [131, 132].

### 9.8 Therapeutic Translocation of Mitochondrial Dysfunction in Neurodegenerative Illnesses: Overview and Future Projections

An important aspect in the pathophysiology of Parkinson's disease (PD) is mitochondrial dysfunction, especially oxidative stress and impaired mitophagy, and perturbed energy metabolism. As it has been shown in this section, the ability to repair mitochondrial activity is a complex yet potentially effective method of improving the disease progression rates or halting them altogether. Mitochondrial antioxidants such as MitoQ, SkQ1, and ALA reduce the number of reactive oxygen species (ROS) in PD models and prevent mitochondrial membranes, proteins, and DNA oxidative damage. Mitochondrion deficits should be removed by improving mitophagy, especially by promoting the use of the PINK1-Parkin pathway, which helps eliminate defective mitochondria and avoids their harmful growth. Small compounds that stimulate mitochondrial turnover/clearance, such as urolithin A, spermidine, resveratrol and NR have shown positive effects in experimental PD systems. To improve mitochondrial bioenergetics, you can maintain ATP levels and reduce neuronal stress by taking metabolic modulators (*e.g*., CoQ10 and NMN) and adjusting your lifestyle (*e.g*., exercise and diet) to change mitochondrial bioenergetics. The conglomeration of these plans marks the shift in the treatment of the parkinson disease, where both methods focus on treating the symptoms of the disease but not the disease itself.

It is interesting to note that the mitochondrial dysfunction is not a specific phenomenon of Parkinson's disease (PD); on the contrary, it is one of the typical clinical features of other neurodegenerative conditions, like amyotrophic lateral sclerosis (ALS) and Alzheimer's disease (AD). Just as is the case with Parkinson's disease (PD), mitochondrial bioenergetic issues, excessive ROS, and poor mitophagy all cause synaptic and neuronal failure in Alzheimer's disease (AD). ALS is characterized by fragmentation of the mitochondria, loss of efficiency of oxidative phosphorylation and loss of calcium handling which worsens the degeneration of motor neurons. The overlap demonstrates that by concentrating on the mitochondrial pathways in various neurodegenerative diseases, the creation of broad-spectrum therapies that act against common pathogenic processes, might be achieved. Nevertheless, there remain translation issues, such as long-term safety profiles, patient diversity and delivery of drugs to the brain. Individualised treatment of neurodegeneration PPP Precision medicine strategies which combine personalised mitochondrial therapy with genetic screening (ex, APP/PSEN mutations in Alzheimer's, PINK1/Parkin mutations in Parkinson) have high potential. We need to learn how mitochondrial dynamics, energy production, and neuronal survival interact to develop effective and long-lasting treatments.

### CONCLUSIONS AND FUTURE DIRECTIONS

Mitochondrial impairment has now been well recognized as playing a major role in the pathogenesis of several neurodegenerative diseases, especially Parkinson's disease (PD), where oxidative phosphorylation represents a strong energy source needed by dopaminergic neurons. There has been excessive study on the neuronal mitochondria, as opposed to the glial mitochondria, which are also relevant in ensuring the neurons are healthy and strong. This review has highlighted the importance of the glial mitochondrial functioning to explain that they are involved in metabolism, inflammation, management of oxidative stress, calcium signalling and control of apoptosis. More recent findings have shown that glial cells are capable of ingesting and recycling damaged neuronal mitochondria, so there may be new therapeutic opportunities.

Mitochondrial dysfunction in Parkinson's disease (PD) is typified by impaired mitophagy, increased reactive oxygen species (ROS) development and reduced ATP production. The mutations in genes such as PINK1, Parkin, DJ-1 and LRRK2 render the mitochondria even less stable and this induces issues with synapses in the brain and persistent inflammation of the brain. Notably, impaired glial mitochondria resilience to protect and sustain the neurons makes the neurodegeneration more enhanced. The complex nature of the interaction between neurons and glial mitochondria in Parkinson's disease has not been fully understood, in spite of massive progress in the field of study. A wide-ranging treatment strategy, which safeguards the well-being of neuronal and glial mitochondria, can be more effective in the future than concentrating on a single type of mitochondria. With the help of effective brain-delivery systems, early intervention can significantly improve the results of the illness. The further research must focus on mitophagy, mitochondrial biogenesis, and dynamics, particularly, the processes that are controlled by the PINK1-Parkin pathway. Targeting glia therapeutics, mitochondrial biomarkers to detect disease earlier, and being able to edit the genome *via* CRISPR, stem cell therapies, and transfer of mitochondria into diseased cells are all promising new concepts. Patient-derived iPSC models have been found to be more and more applicable in developing medicines that are specific to an individual. To sum it up, the preservation and regeneration of the mitochondrial health of the neurons and glia is a powerful plan to slow down the disease process and potentially change the direction of Parkinson’s Disease. The enhancement of knowledge about these interactions will be of high significance to create long-lasting and more effective medicines.

## Figures and Tables

**Fig. (1):**
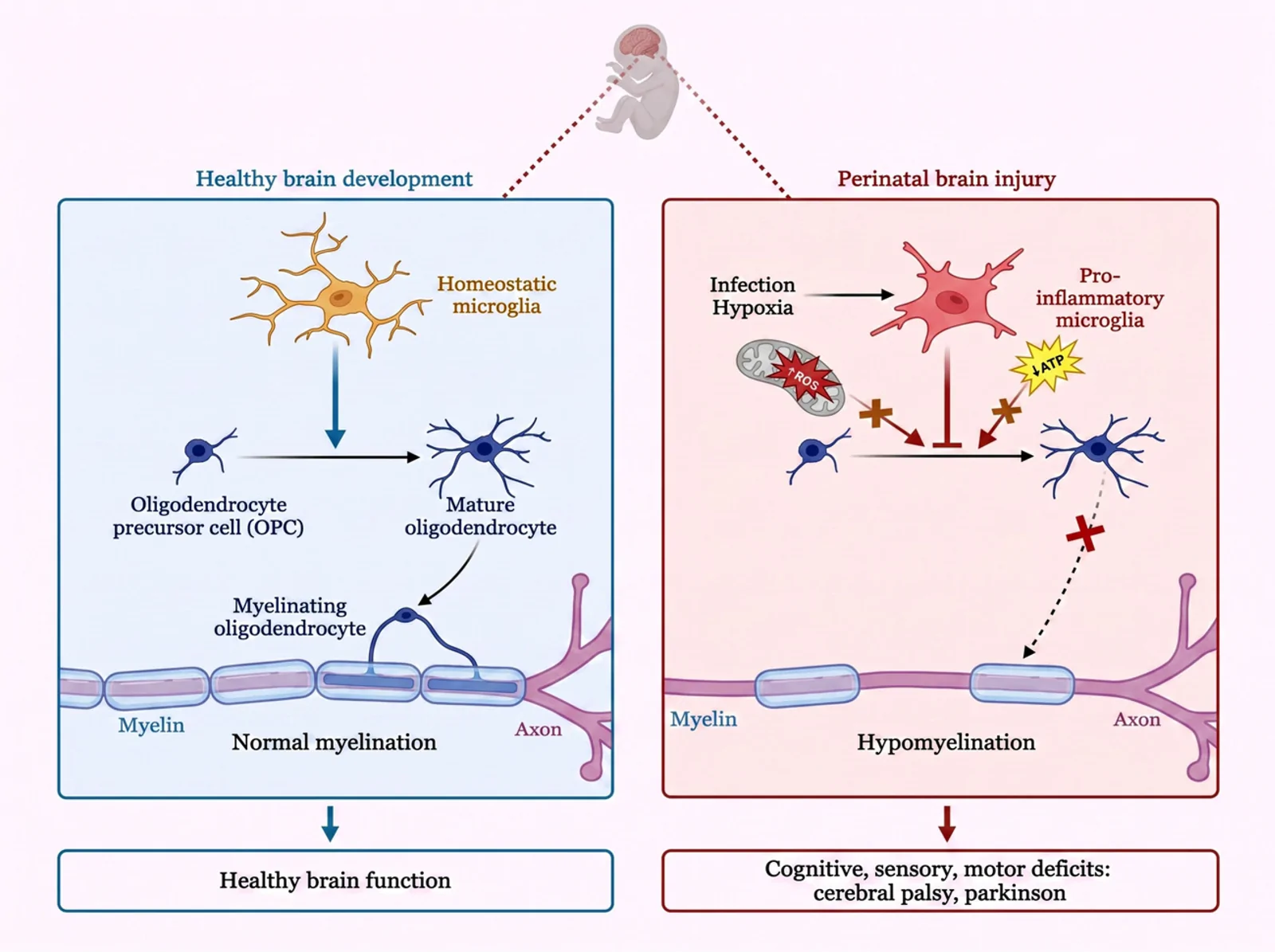
Implications of microglial activity on brain function and myelination during development.

**Fig. (2):**
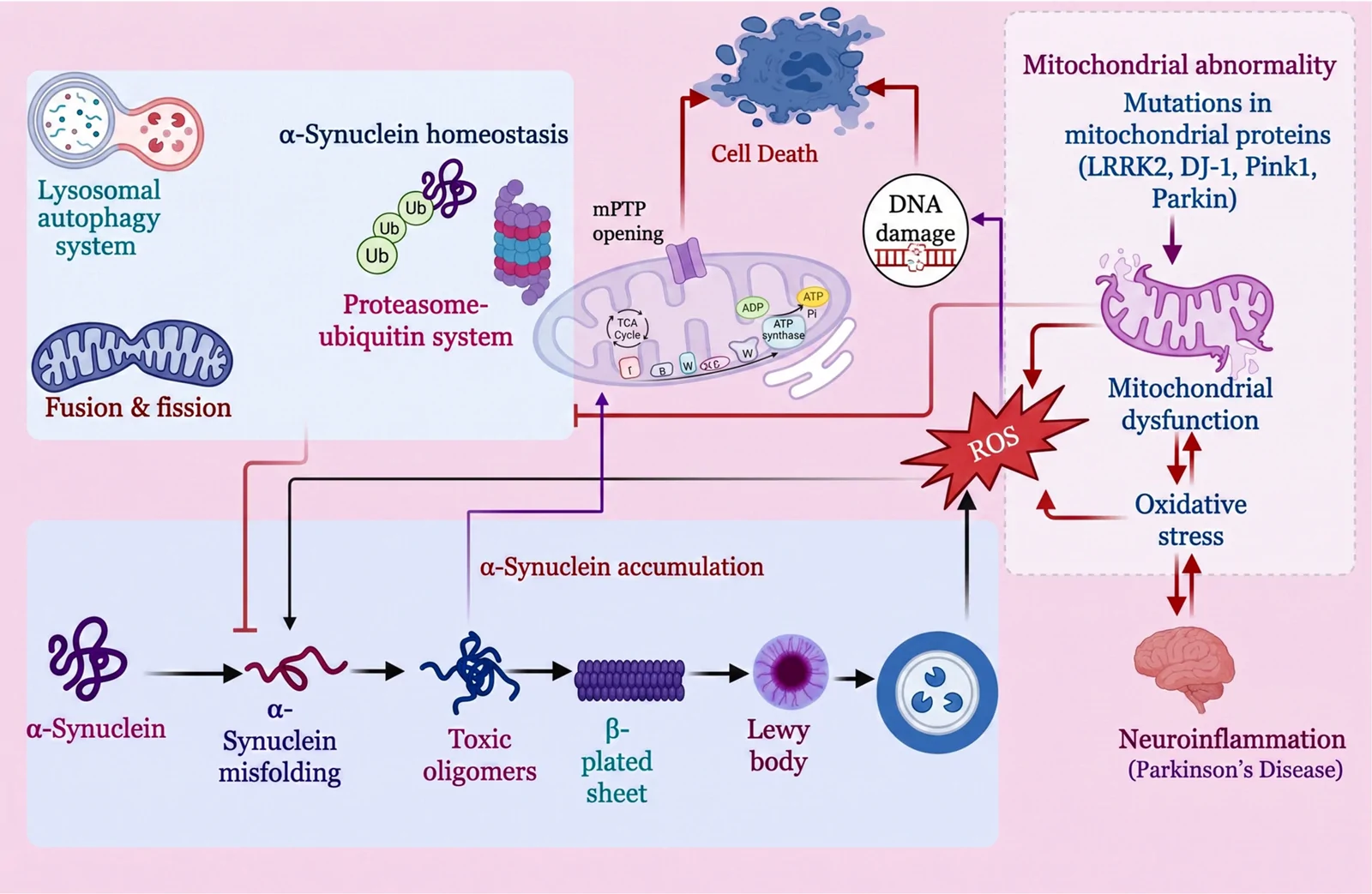
Neuroinflammation, mitochondrial dysfunction, and α-synuclein misfolding in Parkinson's disease.

**Fig. (3):**
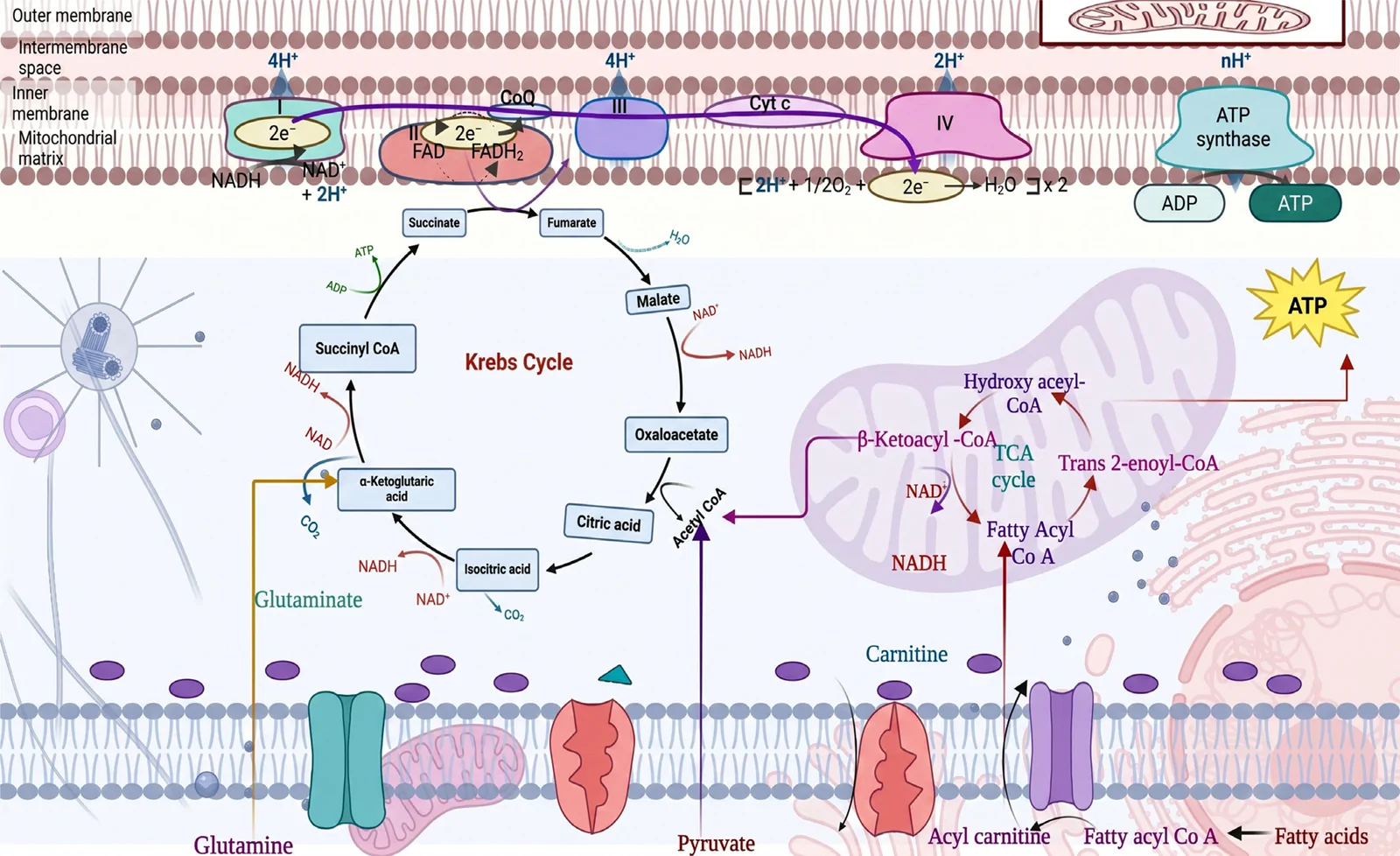
Mechanisms of cellular respiration and mitochondrial ATP generation.

**Fig. (4):**
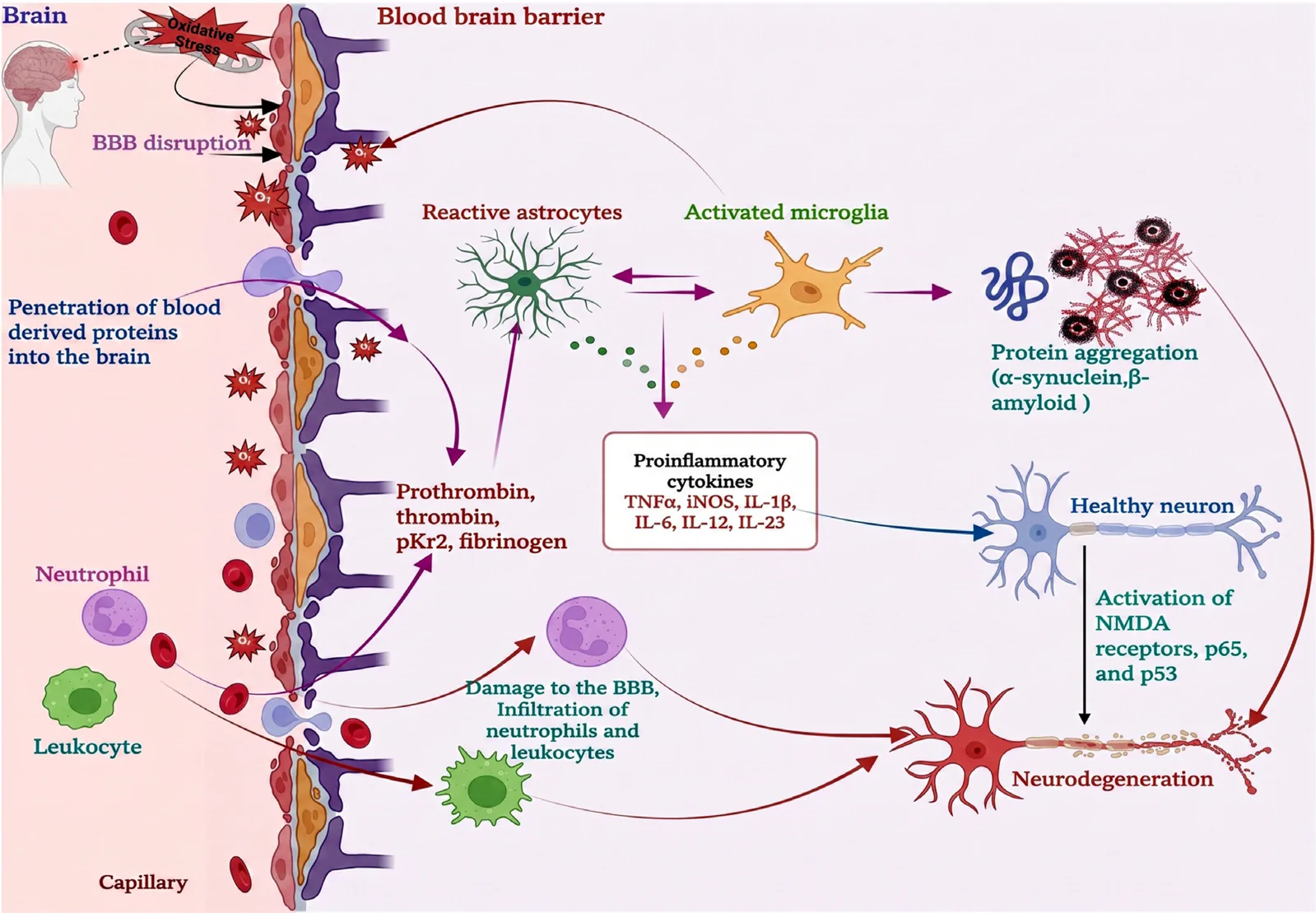
Neuroinflammation-induced neurodegeneration and blood-brain barrier destruction.

**Fig. (5):**
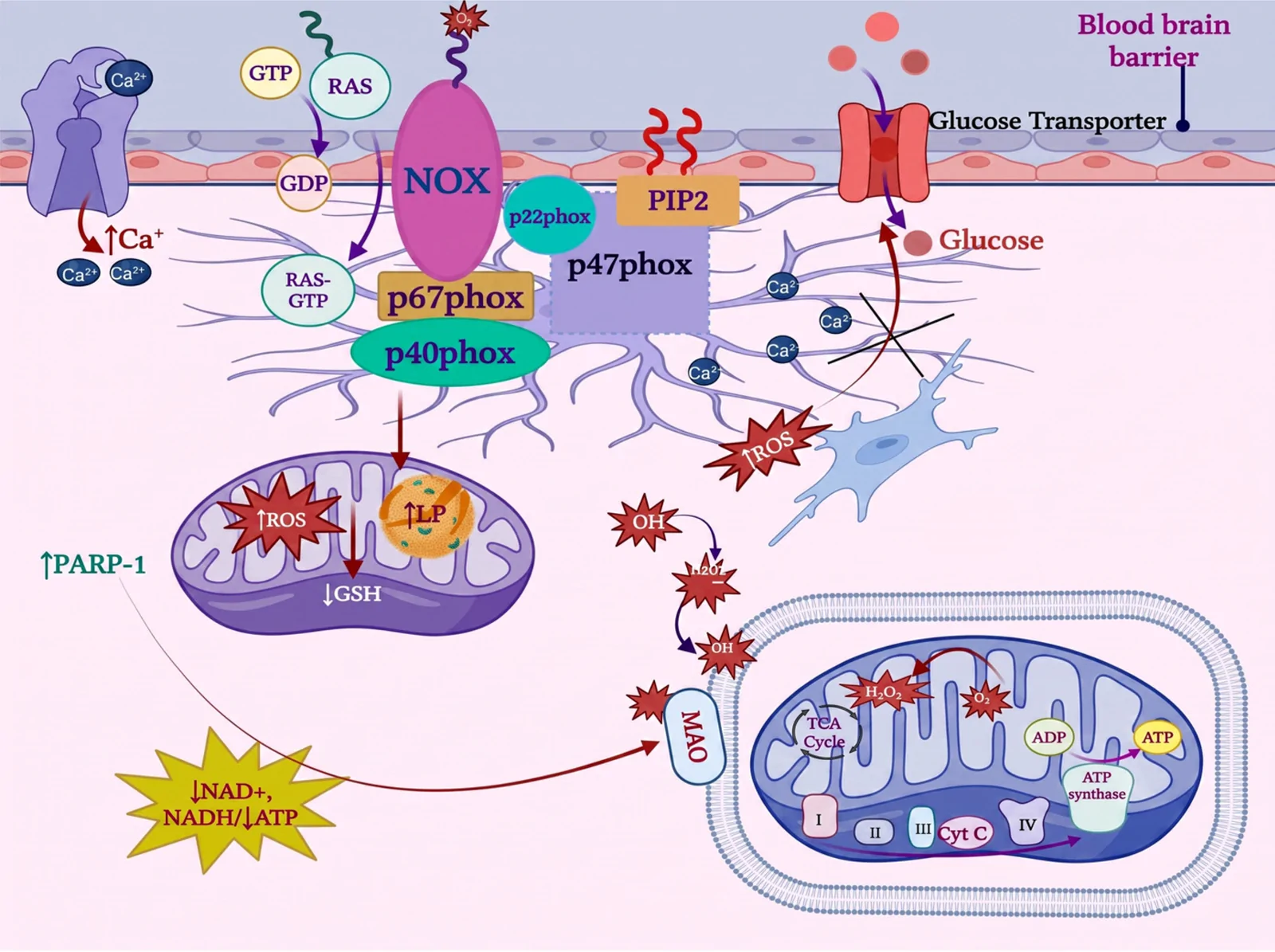
NADPH oxidase and glucose transport through the blood-brain barrier mediate oxidative stress and mitochondrial dysfunction.

**Table 1:** Key factors in neurodegenerative diseases and glial mitochondrial dysfunction.

**Glial OXPHOS Degeneration**	**Role of Crucial Markers**	**Determinants of Glial Dysfunction and Neurodegeneration**
Impaired glutamate aspartate transporter (GLAST/EAAT1) function	The main excitatory neurotransmitter in the CNS is glutamate, released by presynaptic terminals, which diffuses across the synaptic cleft and attaches to receptors. Once EAATs take up synaptic glutamate, glutamatergic transmission stops. Energy generation, modulation of glutamate transmission, prevention of excitotoxicity, and supply of glutamate to neighboring neurons *via* glutamine conversion are all functions of glutamate absorption by EAAT2 [75].	The control of synaptic activity and plasticity depends on EAAT2. EAAT2 links to many central nervous system illnesses. Glutamate excitotoxicity, caused by impaired function of the glutamate aspartate transporter (GLAST/EAAT1), leads to neuronal overstimulation, calcium overload, mitochondrial stress, and neurodegeneration in ALS, epilepsy, and AD [75].
Cytochrome C oxidase (COX) deficiencies	In the electron transport chain, cytochrome oxidase plays a crucial regulatory role. Multiple levels of mitochondrial activity and content regulation demonstrate their relevance. Any of these regulatory trigger disruptions may cause disease [76].	Defective cytochrome oxidase activity impacts many mitochondrial functions; many disorders are linked to CcO deficiency. CcO deficit affects calcium homeostasis and aerobic ATP production. CcO contributes to elevated mitochondrial ROS [76].
Proton-pumping NADH: ubiquinone oxidoreductase (Complex I)	The biggest and most complex respiratory chain enzyme, Proton-pumping NADH: ubiquinone oxidoreductase (complex I), performs NADH oxidation, ubiquinone reduction, and proton pumping. Additionally, the mitochondrial enzyme contains 30 auxiliary subunits surrounding the core subunits, which do not participate in energy conservation [77].	Several inherited and degenerative disorders have been associated with complex I malfunction, which is known to emit harmful oxygen radicals (ROS). Complex I dysfunction manifests as NDUFS1 and NDUFS3 in MS, PD, and ALS. This dysfunction results in oxidative damage, more ROS, lower ATP, and poor glial cell neuroprotection [77].
Ubiquinol-cytochrome C reductase (Complex III)	Ubiquinol-cytochrome C reductase, or Complex III, is essential to the mitochondrial respiratory chain. It enables electron transport from coenzyme Q (ubiquinol) to cytochrome c and pumps protons across the inner mitochondrial membrane to provide the electrochemical gradient for ATP generation [78].	Numerous neurological conditions are Various neurodegenerative illnesses are linked to mitochondrial complex III dysfunction, which impairs energy generation, increases oxidative stress, and causes neuronal damage and death [78].
Cytochrome C Oxidase (Complex IV)	Cytochrome c oxidase, or Complex IV, transfers electrons to oxygen and generates ATP in the ETC [79].	Reduced ATP generation, oxidative stress, and brain damage are linked to mitochondrial complex IV malfunction, notably cytochrome c oxidase, in neurodegenerative illnesses including Alzheimer's, Parkinson's, and Huntington's [79].
ATP synthase (Complex V)	The mitochondrial ETC enzyme complex V, ATP synthase, is essential for ATP production [80].	Due to inadequate energy generation and oxidative stress, mitochondrial malfunction, especially Complex V (ATP synthase), is linked to neurodegenerative illnesses, including Alzheimer's, Parkinson's, and Huntington's [80].

**Table 2:** Critical regulators of mitophagy and associated alterations in PD.

**Mitophagy Type**	**Protein Name**	**Protein Alteration**	**Mechanism/Alteration Outcomes**	**References**
Parkin-Dependent Mitophagy	PINK1	Mutations (*e.g*., PARK6)	Accumulation at OMM (Outer Mitochondrial Membrane), failure to recruit Parkin, impaired mitophagy, mitochondrial dysfunction	[87]
Parkin-Dependent Mitophagy	Parkin	Mutations (*e.g*., PARK2)	Damaged mitochondria accumulate and cause neurodegeneration due to impaired ubiquitination.	[88]
Receptor-Mediated Mitophagy	BNIP3/NIX	Dysregulation	A buildup of damaged mitochondria, decreased LC3 binding, and reduced mitophagy starts.	[89]
Receptor-Mediated Mitophagy	FUNDC1	Altered phosphorylation	Inability to react to mitophagy produced by low oxygen levels, a malfunction in mitochondria	[90]
Parkin-Independent Mitophagy	LRRK2	Mutations (*e.g*., G2019S)	Mitophagy efficiency changes, mitochondrial stress, and RAB10-OPTN signaling dysregulation	[91]
Parkin-Independent Mitophagy	α-Synuclein (SNCA)	Overexpression/Mutation (PARK1)	Misfolding of mitochondria, buildup of mitochondrial debris, and suppression of mitophagy	[92]
Parkin-Independent Mitophagy	DJ-1	Mutations (*e.g*., PARK7)	Elevated free radicals, impaired mitochondrial detoxification	[93]
Parkin-Independent Mitophagy	GBA1	Mutations	Problems with mitophagy, lysosomal dysfunction, and α-synuclein buildup	[94]

**Table 3:** Mechanisms and outcomes of mitochondrial antioxidants.

**Antioxidant**	**Mechanism of Action**	**Outcomes**
Superoxide Dismutase (SOD)	Converts superoxide radicals (O_2_•⁻) into hydrogen peroxide (H_2_O_2_)	Reduces oxidative damage, prevents mitochondrial dysfunction
Catalase	Breaks down H_2_O_2_ into water and oxygen	Protects against hydroxyl radical formation
Glutathione (GSH) System	Neutralizes ROS, regenerates oxidized glutathione (GSSG)	Maintains redox homeostasis, reduces lipid peroxidation
Thioredoxin (Trx) & Peroxiredoxin (Prx)	Reduces oxidative stress by maintaining redox balance	Supports DNA repair, prevents apoptosis
Coenzyme Q10 (CoQ10)	Scavenges ROS, supports ETC	Enhances ATP production, protects cardiovascular health
Alpha-Lipoic Acid (ALA)	Regenerates antioxidants (GSH, vitamins C & E), reduces ROS	Improves insulin sensitivity, protects neurons
MitoQ	Targets mitochondria, neutralizes ROS at the source	Reduces oxidative stress in neurodegenerative diseases
SkQ1	Protects mitochondrial membranes, reduces lipid peroxidation	Delays aging, improves eye and brain health
Resveratrol	Activates sirtuins, enhances mitochondrial biogenesis	Improves metabolic function, extends cellular lifespan

## LIST OF ABBREVIATIONS

AD: Alzheimer's Disease
ALS: Amyotrophic Lateral Sclerosis
AMPA: α-Amino-3-hydroxy-5-Methyl-4-Isoxazolepropionic Acid
ASC: Apoptosis-associated Speck-like Protein Containing a CARD
ATP: Adenosine Triphosphate
BBB: Blood-Brain Barrier
CNS: Central Nervous System
DA: Dopaminergic
DJ-1: Protein Deglycase DJ-1
DRP1: Dynamin-Related Protein 1
ER: Endoplasmic Reticulum
ETC: Electron Transport Chain
IL-1β: Interleukin 1 Beta
IL-6: Interleukin 6
iNOS: Inducible Nitric Oxide Synthase
LRRK2: Leucine-Rich Repeat Kinase 2
MAO-B: Monoamine Oxidase B
MCU: Mitochondrial Calcium Uniporter
Mfn: Mitofusin
MFN1/2: Mitofusin 1 / 2
MPP+: 1-Methyl-4-Phenylpyridinium
MPTP: 1-Methyl-4-Phenyl-1,2,3,6-Tetrahydropyridine
mtDNA: Mitochondrial DNA
NLR: NOD-Like Receptors
NLRP3: NOD-, LRR- and Pyrin Domain-containing Protein 3
NMDA: N-Methyl-D-Aspartate
Nrf2: Nuclear Factor Erythroid 2-related Factor 2
OPA1: Optic Atrophy 1
OXPHOS: Oxidative Phosphorylation
PARKIN: E3 Ubiquitin-Protein Ligase Parkin
PD: Parkinson's Disease
PINK1: PTEN-Induced Kinase 1
ROS: Reactive Oxygen Species
SN: Substantia Nigra
SNpc: Substantia Nigra Pars Compacta
SOD: Superoxide Dismutase
TIM: Translocase of Inner Membrane
TNF-α: Tumor Necrosis Factor Alpha
TOM: Translocase of Outer Membrane
UCP2: Uncoupling Protein 2
UPS: Ubiquitin Proteasome System
VDAC: Voltage-dependent Anion Channel
α-syn: Alpha-synuclein
